# *In Silico* Design of Molecularly Imprinted Poly(*o-*aminophenol) for Melamine and Cyanuric Acid Detection and Extraction

**DOI:** 10.1021/acsmeasuresciau.6c00116

**Published:** 2026-07-06

**Authors:** Enayat Mohsenzadeh, Vilma Ratautaite, Agne Ramanaviciute, Krzysztof R. Noworyta, Arunas Ramanavicius

**Affiliations:** † Department of Nanotechnology, State Research Institute Center for Physical Sciences and Technology (FTMC), Sauletekio Ave. 3, LT-10257 Vilnius, Lithuania; ‡ Department of Physical Chemistry, Institute of Chemistry, Faculty of Chemistry and Geosciences, Vilnius University (VU), Naugarduko Str. 24, LT-03225 Vilnius, Lithuania; § Department of Physics, University of Cambridge, Cavendish Laboratory, JJ Thomson Avenue, Cambridge CB3 0HE, U.K.; ∥ Institute of Physical Chemistry, Polish Academy of Sciences, Kasprzak 44/52, 01-224 Warsaw, Poland

**Keywords:** density functional theory (DFT-D4), molecularly imprinted polymer (MIP), poly(o-aminophenol) (PoAP), melamine (MEL), cyanuric acid (CYA), bisphenol A

## Abstract

Melamine (MEL) is a heterocyclic aromatic compound that is carcinogenic to humans and causes damage to organs through prolonged or repeated exposure. It is typically used in the resin-coating industry as a cross-linker to produce durable plastic packaging. Molecularly imprinted polymer (MIP)-based structures seem very promising for the detection/extraction of some organic compounds. Therefore, in this study, density functional theory (DFT-D4) is used to guide the *in silico* design of an MIP suitable for the detection/extraction of melamine and cyanuric acid. The MIP preparation steps, including analysis of candidate monomers, analysis of the oligomer’s oxidation state and flexibility, optimization of prepolymer complex composition, formation of binding sites, and studies of the analytes’ rebinding, are evaluated. Three monomers (aniline (ANI), *o*-phenylenediamine (oPD), and *o*-aminophenol (oAP)) and their corresponding oligomers are evaluated to select the optimal receptor for the target analytes based on their binding energies, hydrogen bonding, electrochemical properties, and electrostatic potential maps. The obtained values indicate a higher electron affinity for poly­(*o*-aminophenol) (PoAP). The analysis of conformations also reveals greater flexibility in PoAP, which allows for more efficient formation of imprinted cavities. Moreover, the effect of the solvent on the hydrogen bonding and binding energies is screened. Dimethyl sulfoxide (DMSO) is the best solvent, showing minimal interference. The modeled MIP’s binding sites demonstrate the role of homogeneous cavity formation in achieving improved selectivity, characterized by the higher specific noncovalent interactions attained by imprinted PoAP. A nonimprinted polymer (NIP) is modeled as a reference to evaluate imprinting efficiency. Accordingly, approximate imprinting factors (IF) of 2.71 for melamine and 1.91 for cyanuric acid are obtained.

## Graphical abstract

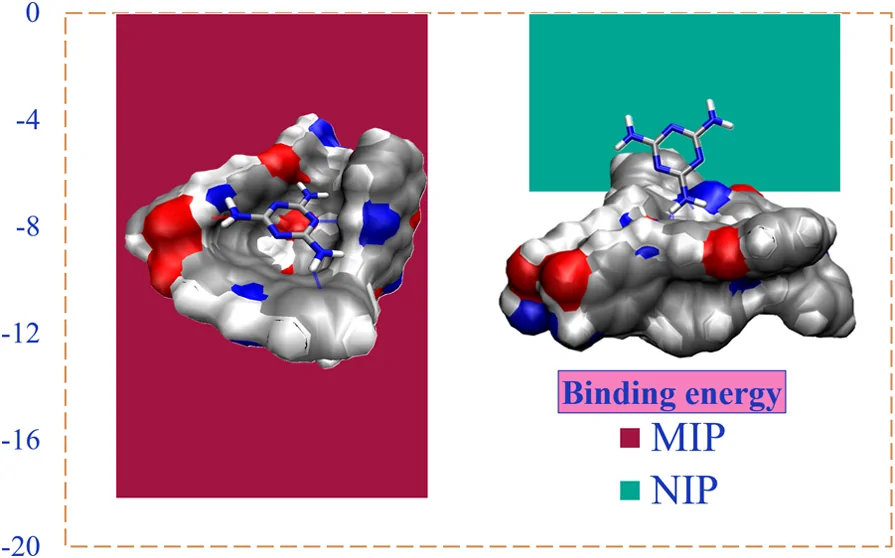

## 1 Introduction

Computational methods (e.g., molecular mechanics (MM), molecular dynamics, Monte Carlo simulations, quantum mechanics, and statistical simulations) are widely used to guide experimental design, thereby reducing reliance on costly trial-and-error experiments or to analyze, rationalize, and confirm experimental findings.^1,2^

In this computational study, density functional theory (DFT) was employed to evaluate the molecularly imprinted poly­(*o*-aminophenol) (PoAP)-based sensor for melamine (MEL) and cyanuric acid (CYA). The interactions between the MIP and the analytes (MEL and CYA) were modeled using DFT, extended tight-binding (GFN2-xTB), and MM (MMFF94s) to investigate the MIP’s imprinting process and its subsequent binding interactions.

Melamine (MEL), selected as one of the target analytes for this study, is a heterocyclic aromatic compound with 66% nitrogen by mass, which is “carcinogenic to humans” (Carc. 2, H351) and “may cause damage to organs through prolonged or repeated exposure” (STOT RE 2, H373).^3^ It is typically used in the resin-coating industry as a cross-linker to produce durable plastic packages.^4^ MEL has garnered significant public attention due to controversies surrounding its use as an adulterant in sports nutrition,^5^ infant powder,^6^ dairy products, and animal foods.^7,8^ Additionally, it has been demonstrated that MEL can migrate from tableware containing MEL formaldehyde into food.^9^ Cyanuric acid (CYA) is the main byproduct of MEL production and is detected in the adult population in the U.S. simultaneously with melamine.^10^ Besides, CYA is widely used for chlorine stabilization, where chlorination is a common way to ensure the safety of recreational waters in recirculation systems, including swimming pools, thereby posing threats to water resources.^11^

Numerous studies have demonstrated that MEL has a diverse range of harmful effects on human health. Rajpoot et al.^12^ have highlighted the importance of better MEL testing due to its association with toxicity to the gut microbiota. Hau et al.^13^ have identified that high dosages of MEL and CYA intakes cause urinary stones, crystalluria, and acute renal failure in humans and animals. Lütjens et al.’s^14^ study reviewed the data for MEL and CYA presence in the environment. They found several cases of scarcity of measurements. Analysis of the limited data available indicates the presence of MEL and CYA in the surface water from various locations. Sun et al.^15^ have reported that the copresence of these two compounds exhibits a synergistic neurotoxic effect resulting in cell apoptosis and disturbed energy metabolism.

Various analytical methods have been developed to detect MEL reliably and accurately. These methods include Raman spectroscopy,^16,17^ UV–Vis spectroscopy,^18^ enzyme-linked immunosorbent assay (ELISA),^19^ and high-performance liquid chromatography (HPLC).^20,21^ Paranthaman et al.’s^22^ study used X-ray diffraction for the simultaneous detection of MEL and CYA. Although these methods are reliable, they are costly and operationally intensive. Therefore, developing an on-site and inexpensive sensor to detect these compounds has notable advantages.

Molecularly imprinted polymers (MIPs) are artificial receptors with binding sites specific to targets.^23^ MIPs are synthesized by the polymerization of a functional monomer(s) in the presence of the target molecule known as the template. After polymerization, the removal of the template creates an imprinted polymer containing complementary binding sites.^24,25^ This feature enables MIPs to possess higher selectivity and sensitivity to the target molecules than the nonimprinted polymers (NIPs).^26^ Several factors determine the MIP’s performance. The main parameters are (i) the optimal functional monomer, (ii) the monomer-to-template molar ratio, (iii) the corresponding polymer structure,^27^ (iv) the medium (solvent type and pH),^28^ and (v) the effectiveness of the template extraction.^29^

Several studies have investigated MIPs for MEL detection using both experimental and computational approaches. Brazys et al.^30^ synthesized a polypyrrole-based MIP for MEL detection. In addition, the study modified the electrochemical sensor with gold nanoparticles to increase its sensitivity. Pietrzyk et al.^31^ developed an acoustic chemosensor based on the MIP for MEL. They used DFT calculations to visualize the complex systems. Bates et al.^32^ used molecular dynamics (MD) simulations to reduce the experimental trials and fabricate the MIP for MEL recognition. Biabani et al.^33^ used statistical methods in their experiments to optimize the synthesis of MIPs for the electrochemical detection of MEL. However, it must be noted that in these studies, MEL and CYA are not considered for simultaneous detection.

Computational methods are perceived as a green approach to avoid tedious and chemical-consuming trial-and-error experiments, while obtaining important information about the analyzed system. They facilitate studies of interactions and the selection of the optimal configuration of chemicals.^34^ Furthermore, they allow for a deeper understanding of the experimental results at the molecular level. Because of these advantages, computational methods, such as DFT, have been extensively used to model and design a variety of MIPs.^2,35^ Rebelo et al.^36^ used a rational computational strategy to develop an MIP-based sensor for detecting furazolidone (a nitrofuran antibiotic). The authors first performed DFT calculations to screen and identify the most suitable functional monomer by evaluating the binding energies between furazolidone and several candidate monomers. After selecting the optimal monomer, they conducted MD simulations of the prepolymerization mixture to better understand the template–monomer interactions in solution. In a subsequent study, Rebelo et al.^37^ used a similar approach to rationally design MIP-based electrochemical sensors for the detection of atorvastatin. DFT calculations guided monomer selection based on binding energies, while MD simulations provided insights into prepolymerization interactions via radial distribution functions (RDFs) and H-bond analyses. Xie et al.’s^38^ study focuses on an MIP-based morphine sensor. The study employed molecular electrostatic potential (MEP) maps, frontier molecular orbital analysis, and the interaction region indicator (IRI) to visualize and characterize key interaction sites, thereby aiding in identifying the optimal prepolymerization system. Their computational strategy included a conformational search to identify the most stable configurations, followed by geometry optimization using the semiempirical GFN2-xTB method and subsequent refinement with DFT calculations.

The novelty of this study lies in the rational application of *in silico* methods to evaluate the influence of key parameters governing the MIP performance. This includes the prepolymerization mixture composition (the most suitable monomer, the optimal monomer-to-template ratio, and the solvent choice) for the prepolymerization complex. This study also aims to investigate the influence of oligomer configurations and conformations on complex formation, an aspect frequently overlooked in MIP computational studies. Moreover, we investigate the interactions of the simulated imprinted binding sites and selected analytes in more detail to determine which type of binding is prevalent. In addition, we examined the effect of the cavity structure on the binding of the analytes and interferences. The optimized conditions identified in this work facilitate the rational design of more efficient imprinted polymer receptors. Scheme 1 illustrates the strategies and analyses used in this work.

**Scheme 1:**
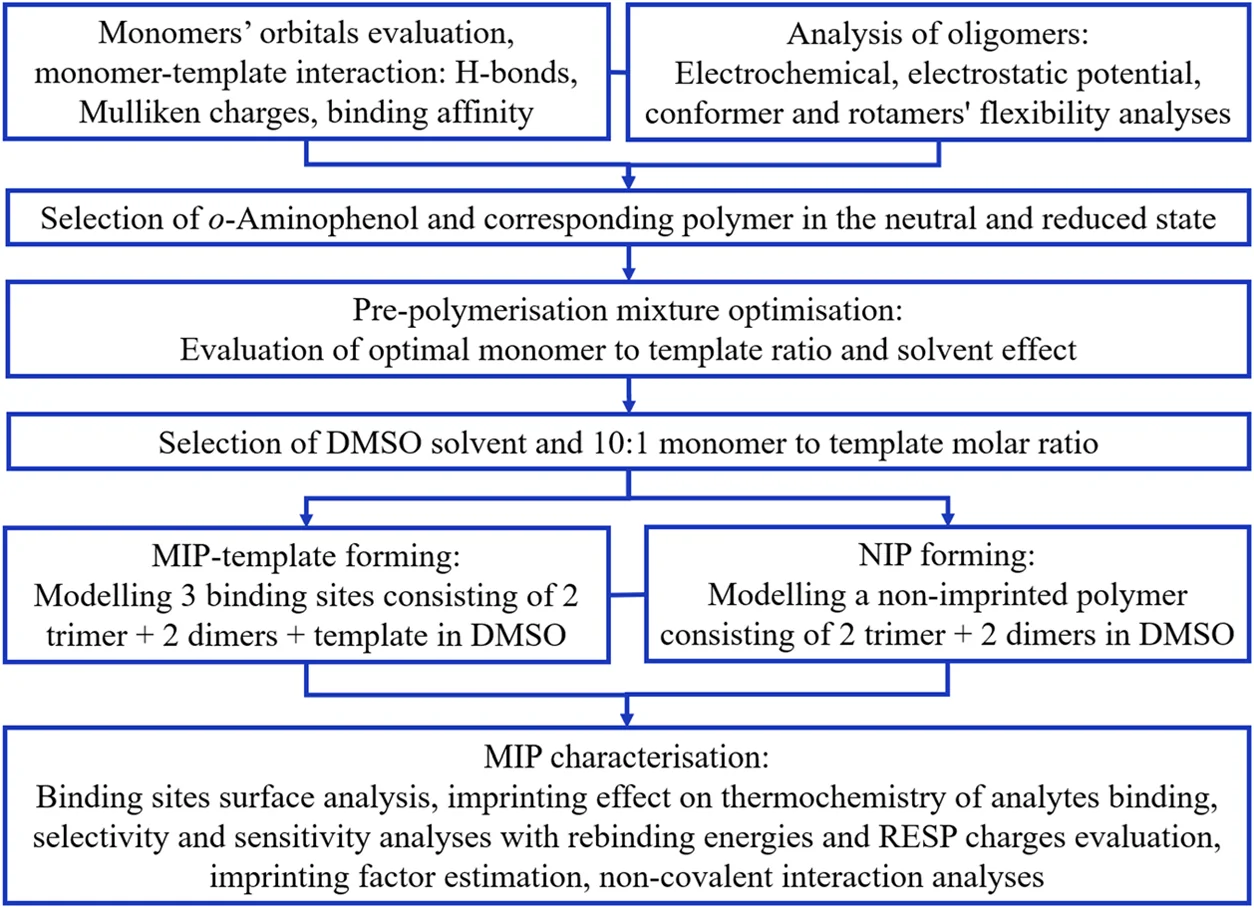
Summary of the Computational Steps Employed for the *In Silico* Design of the Molecularly Imprinted Sensor Based on Poly­(*o*-aminophenol) (PoAP) for Melamine (MEL) and Cyanuric Acid (CYA) Detection

## 2 Results and Discussion

### 2.1 Analysis of Candidate Monomers

Molecular orbital (MO) theory describes the molecular structure, electron density, and reactivity of molecules. Orbitals of the aromatic bonds are presented in two mathematically equivalent shapes if they are not perturbed. The HOMOs are known as the electron donor, and the LUMOs are known as the electron acceptor. The electrons of these orbitals are most accessible to electrophiles and nucleophiles. The functional aromatic structures of the candidate monomers aniline (ANI), *o*-phenylenediamine (oPD), and *o*-aminophenol (oAP) are depicted in Figure 1. The results show comparable frontier orbitals and gap energy values for all three monomers. However, the presence of an adjacent functional group at the ortho-position in oPD and oAP can induce delocalization of the electron cloud through electronic coupling and orbital perturbation. This effect is particularly evident with an ortho-hydroxyl group, as its highly electronegative oxygen atom forms an intramolecular hydrogen bond with the neighboring amine, which facilitates the delocalization of electron density into an adjacent LUMO. However, two symmetrical amine groups and nonbonding lone pairs in oPD increased both HOMO and LUMO energy levels, as well as the softness of the molecule. This results in oPD having a slightly higher reactivity (a lower *E*_g_ value) with the highest HOMO energy, acting as the best electron donor. oAP showed the lowest HOMO and LUMO energies, representing the weakest electron donor and the best electron acceptor case. Therefore, the global reactivity of these monomers was found to be very close, but in the decreasing order of oPD > ANI > oAP. The amine group is more likely to participate in the polymerization via a reaction with an approaching monomer or oligomer due to its higher electron-donating capability. Thus, the open configuration in the PoAP is more feasible.

**Figure 1:**
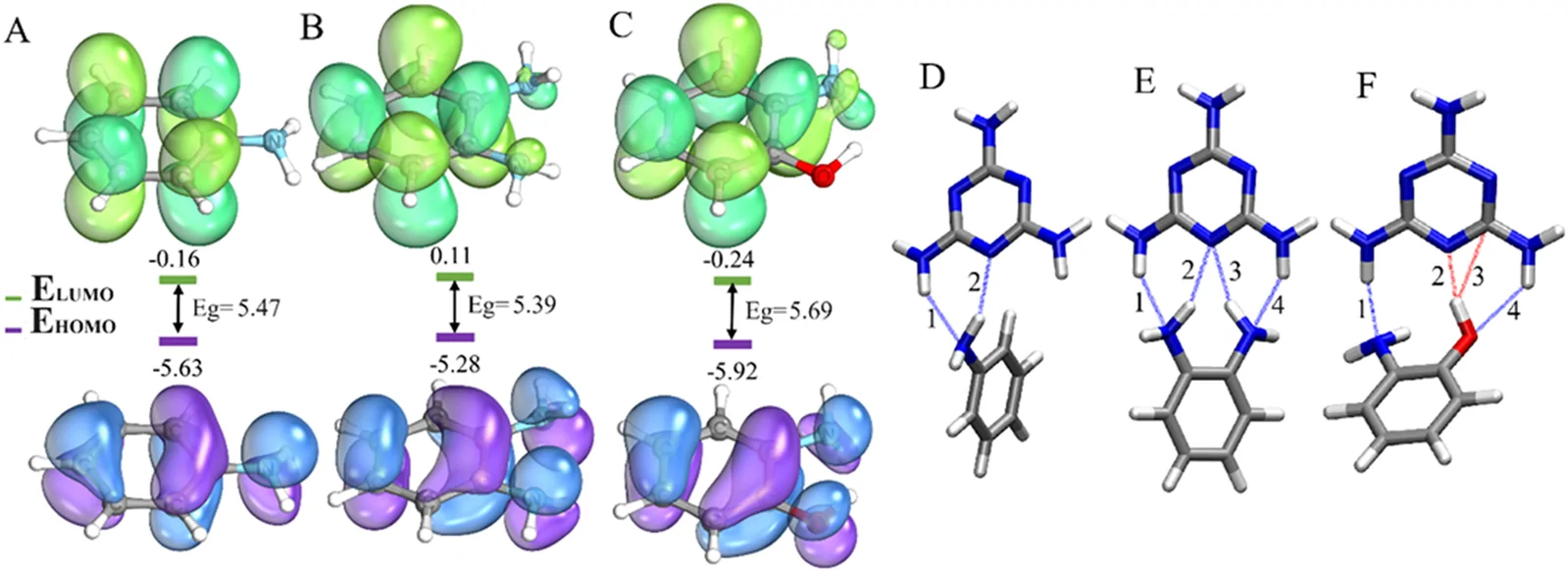
MO of optimized monomers and the corresponding gap energy between the HOMO and LUMO in eV: ANI (A), oPD (B), and oAP (C). H-bond formation between melamine (MEL) and different monomers: ANI-MEL (D), oPD-MEL (E), and oAP-MEL (F) complexes. The intermolecular H-bonds between monomers and MEL are shown by dashed lines in the color of atoms donating hydrogen: blue for the amine functional group and red for the hydroxyl.

CYA is typically mentioned as an interfering molecule rather than as the template. However, CYA is the main metabolic byproduct of MEL and is often co-detected with it in human samples.^10^ Therefore, in this study, MEL is accepted as the template, and the CYA molecule is considered the co-analyte. Three monomer–MEL complexes with the formed H-bonds are presented in Figure 1. The distance (Å) and angle (°) of donor–acceptor bonds (D-H···A) are listed in Table 1 as a measure of the H-bond strength. Bigger angles, ideally 180°, and shorter distances indicate stronger H-bonds. The strength of H-bonds was found to be in a decreasing order of oAP > oPD > AN in the monomer–MEL complexes. The strong H-bond, characterized by 1.69 Å and 164°, between the hydroxyl group of oAP and nitrogen in MEL (Figure 1 (D) H-bond no. 2, Table 1) was found to be the reason for the stronger interaction and increased binding energy.

**Table 1:** Energy of the Monomer–Template Complex Formations and H-Bonds with the Corresponding Distances and Angles between Atoms Involved in H-Bonding and Their Mulliken Atomic Charges

complex	Δ*E*_Binding_ (kcal·mol^–1^)	H-bonds No.	H···A distance (Å), D-H···A angle (deg)	Mulliken gross atomic charges (Donor, H, Acceptor)
ANI-MEL	–8.62	1	2.27, 148	N: –0.36, H: +0.29, N: –0.60
		2	2.17, 150	N: –0.60, H: +0.32, N: –0.34
oPD-MEL	–13.22	1	2.37, 142	N: –0.36, H: +0.24, N: –0.59
		2	2.11, 149	N: –0.59, H: +0.26, N: –0.34
		3	2.20, 142	N: –0.52, H: +0.25, N: –0.34
		4	2.30, 144	N: –0.36, H: +0.25, N: –0.59
oAP-MEL	–14.74	1	2.09, 168	N: –0.33, H: +0.30, N: –0.62
		2	1.69, 164	O: –0.47, H: +0.33, N: –0.26
		3	2.55, 135	O: –0.47, H: +0.33, C: +0.17
		4	2.30, 135	N: –0.38, H: +0.28, O: –0.47

Mulliken gross atomic charges of molecules were assessed to analyze the H-bonding interaction strength. Atoms that act as acceptors are expected to exhibit high negative charges, and atoms that act as donors are characterized by lower charges. Meanwhile, hydrogens experience elevated positive values due to being shared with two electron-withdrawing atoms. Compared with ANI, the additional ortho-amine group in oPD increases the total number of hydrogen bonds. However, the same group also induces steric hindrance, which in turn reduces the strength of H-bonds 1 and 2 (Figure 1, Table 1). A substantial difference lies in the H-bonds formed by the hydroxyl functional groups of oAP. One carbon H-bond was also detected. The energies of complex formation, named binding energy, were calculated using eq 1. The results of these calculations are listed in Table 1. The results showed the strongest affinity for the oAP-MEL complex. The ANI-MEL complex had the lowest affinity, followed by the oPD-MEL complex.

### 2.2 Analysis of Oligomers

Depending on the polymerization method and conditions, different polymer structures can be obtained. Researchers have proposed different structures, named ladder (L) and open ring (O), for the PoAP^39–43^ and the PoPD,^44,45^ and an open ring for polyaniline (PANI).^46,47^ The ladder structure is more dominant for PoPD (making a phenazine-like structure) than for PoAP (phenoxazine-like structure).^45,48–50^ Therefore, both “O” and “L” forms of PoAP and PoPD oligomers were modeled. PANI has an open-ring form with an amino group linked to the rings via head-to-tail coupling. For the sake of simplicity in comparison, the study of bond formation via oxidation of oligomers was ignored.

#### 2.2.1 Molecular Electrostatic Potential (MEP) of Oligomers

In these calculations, we assume that the amino and hydroxyl groups in PoAP and the amino groups in PoPD were oxidized via proton-coupled electron transfer. The MEP of oligomers is depicted with a color range of ± 0.07 in Jmol 16.2.17 software. The open PoAP structures possess higher electrophilicity indices than those of PoPD. This finding is confirmed by the electrostatic potential maps, seen in Figure 2, which show more intense blue regions (indicating greater electrophilicity) for PoAP. In comparison, the oxidation of amines reduced the access of electrons, which is indicated by red areas turning yellow via oxidation. Meanwhile, the ether functional group in the PoAP ladder form remained electron-rich, as it did not participate in oxidation and protonation. Protonation of all chains shifted the electron access areas from the edge to the inner planar sides. The expansion of chains is accompanied by changes in protonation in the electrostatic maps. The maps highlighted higher electron access and deficiency regions for PoAP and the lowest for PANI. Blue side edges show the presence of protons and indicate regions with electron deficiency. PANI underwent complete oxidation (pernigraniline base) and protonation (pernigraniline salt). The two ortho-amine groups in oPD can both participate in polymerization, a process which can result in two primary structures—a ladder or an open-ring configuration. These distinct structural motifs impart different electrochemical properties to the resulting PoPD. For instance, in both the ladder and open-ring configurations of PoPD, all primary and secondary amine groups were found to be oxidized. Moreover, the open structure of the protonated PoAP becomes quite planar due to electrostatic repulsive forces.

**Figure 2:**
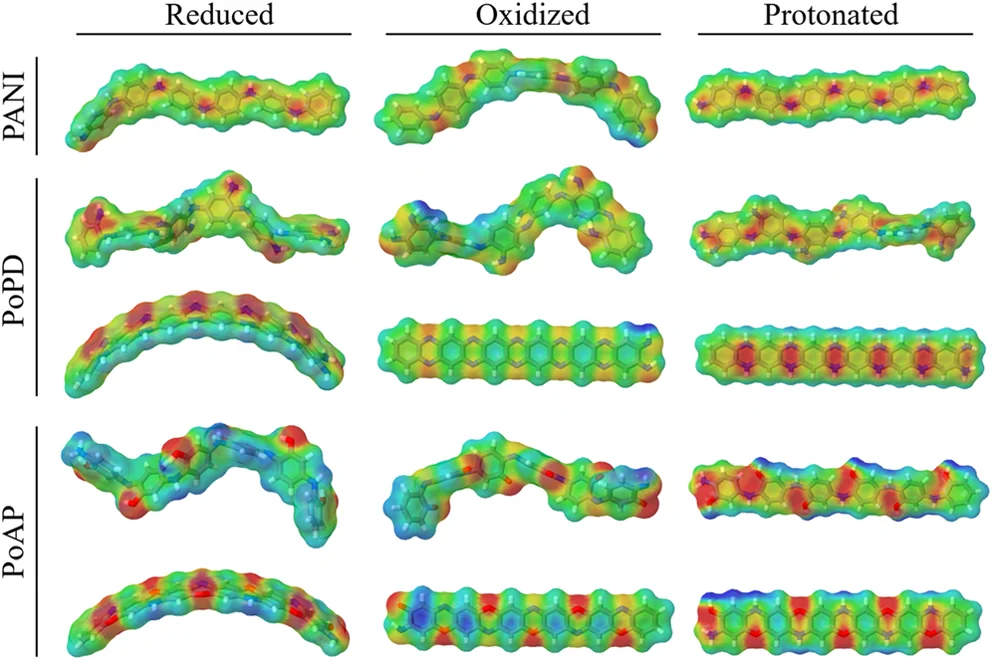
MEP of PANI, PoPD, and PoAP hexamers in three reduced, fully oxidized, and protonated states obtained at the B3LYP/def2-TZVP level. Red color represents regions with electron excess, and blue represents regions with electron deficiency.

#### 2.2.2 Electrochemical Characterization of Oligomers

The electrochemical properties of oligomers (see Figure 2) were calculated using eqs 2–11. The resultant data are shown in Table 2. The oxidation increased ionization potential (IP) and electron affinities (EA). Chemical hardness is equal to half of the energy gap. In their open-chain configurations, the oligomers exhibited a hardness trend of PoAP > PoPD > PANI, while the softness correspondingly followed the inverse order of PANI > PoPD > PoAP. In the case of a more conjugated ladder-type structure, the chemical hardness for PoAP is still higher than that for PoPD. This is due to the more compact electron distribution of oAP, which arises from a higher net nuclear charge. The loss of electrons reduced the molecule’s chemical hardness value. Meanwhile, the increasing softness led to a greater polarizability and reactivity of the oligomers.

**Table 2:** Electrochemical Properties of Oligomers (Hexamers) Derived from the Frontier Orbital Energies (eV) in Reduced and Fully Oxidized States Obtained at the B3LYP/def2-TZVP Level^a^

hexamer	*E*_g_	*IP*	*EA*	*η*	*S*	*χ*	*ω*	*υ*	Δ*N*_max_
PANI	3.83	4.33	0.50	1.91	0.52	2.42	1.53	0.6553	1.26
ox-PANI	2.26	5.56	3.31	1.13	0.89	4.44	8.71	0.1148	3.93
O-PoPD	4.09	4.41	0.32	2.05	0.49	2.37	1.37	0.7302	1.16
L-PoPD	3.30	3.54	0.23	1.65	0.61	1.89	1.08	0.9290	1.14
ox-O-PoPD	2.17	5.98	3.81	1.08	0.92	4.90	11.06	0.0904	4.52
ox-L-PoPD	0.31	5.43	5.12	0.15	6.50	5.27	90.40	0.0111	34.29
O-PoAP	4.85	5.59	0.73	2.43	0.41	3.16	2.06	0.4864	1.30
L-PoAP	3.62	4.18	0.56	1.81	0.55	2.37	1.55	0.6467	1.31
ox-O-PoAP	1.06	5.67	4.61	0.53	1.88	5.14	24.92	0.0401	9.69
ox-L-PoAP	0.92	8.12	7.66	0.46	2.17	7.66	63.62	0.0157	16.60

a Two possible open (O) and ladder (L) configurations were considered for PoAP and PoPD. The prefix “ox” indicates the hexamer oxidation.

The electrophilicity (ω) and nucleophilicity (υ) indices describe the capability of the oligomers to accept and donate electrons, respectively. High values of ω and υ indicate that the oligomers possess high electrophilic and nucleophilic properties. Table 2 illustrates that in the oxidized form, the loss of electrons increased the Mulliken electronegativity (χ), electrophilicity index (ω), and reduced nucleophilicity (υ) index. The amino and hydroxyl groups in PoAP and the amino groups in PoPD were oxidized via proton-coupled electron transfer. This was modeled with hydroxyl and amine groups participating in oxidation, losing an electron and giving away their hydrogen, thereby forming a double bond with the adjacent carbon. The carbonyl groups of PoAP, with an electron deficiency, increased the electron affinity of the polymer. In the ladder configuration, the ether functional groups did not participate in the oxidation. This brought about a lower electrophilic index than the open-ring keto structure.

Open rings of PoPD and PoAP in the oxidized form showed that hydroxyl oxidation (a keto form of PoAP) caused a higher increase in the electrophilicity index (ω) compared with the oxidation of the primary amine group in PoPD. Meanwhile, the absence of the hydroxyl functional group in the ladder form of PoAP (quinoid form) resulted in lower values of ω. The maximum charge transfer signifies the maximum charge the molecule can exchange with its environment. The charge transfer is higher in the ladder form than in the open ring form.

Substantial changes in the electrochemical parameters’ values derived from the HOMO–LUMO show that the complete oxidation of the polymers is unlikely under moderate conditions. MEP analysis displayed that the electron-rich and electron-poor regions were highly perturbed via oxidation and protonation. Therefore, partial oxidation and protonation are expected in the electrochemical processes. This signifies the importance of an open structure, where primary amines and hydroxyl groups are more likely to cause chain folding and flexibility and undergo protonation and deprotonation with increased solubility. Overall, PoAP with the lowest softness has the highest potential due to its lowest reactivity and better chemical stability in the reduced state. It also exhibits higher EA and IP values, which are expected to result in stronger noncovalent interactions.

#### 2.2.3 Flexibility of Oligomers

The flexibility of the polymer chains is a key factor in the imprinting process of a small molecule within the polymeric network, where the electron-withdrawing and electron-donating regions of macromolecules can have the highest accessibility to form binding sites with the highest affinity. Therefore, to ensure the highest flexibility, the polymer chains are modeled in the reduced states. Figure 2 demonstrates that protonation causes repulsive hindrance forces, making the polymer expand and consequently increasing its rigidity. This finding is critical, as the objective of these calculations is to select a polymer with greater ‘flexibility’, which is required to form an efficient imprinted site for small molecules.

Figure 3A shows the average end-to-end distance and Figure 3B shows the corresponding radius of gyration, *R*_g_, for the hexamer conformers with different patterns of alternating ladder (L) and open (O) ring bonding as measures of conformational flexibility. The studied bonding sequences were “OOOOO”, “OOLOO”, “OLOLO”, “LOLOL”, “LLOLL”, and “LLLLL”, where the number of open bonds was five to zero (ladder) in the hexamers, respectively. Figure 3C,3D presents the *R*_g_ distributions for rotamer ensembles of PoAP and PoPD, respectively. PANI was excluded from further analysis as it exhibited the lowest binding energy and the least pronounced electron-rich and electron-poor regions in the previous sections. PoAP exhibits greater overall conformational flexibility, as evidenced by larger variations in both end-to-end distance and *R*_g_ upon introduction of open-ring units. In contrast, PoPD displays relatively smaller changes in these parameters from the fully ladder structure up to three open rings, followed by a marked decrease at higher open-ring content.

**Figure 3:**
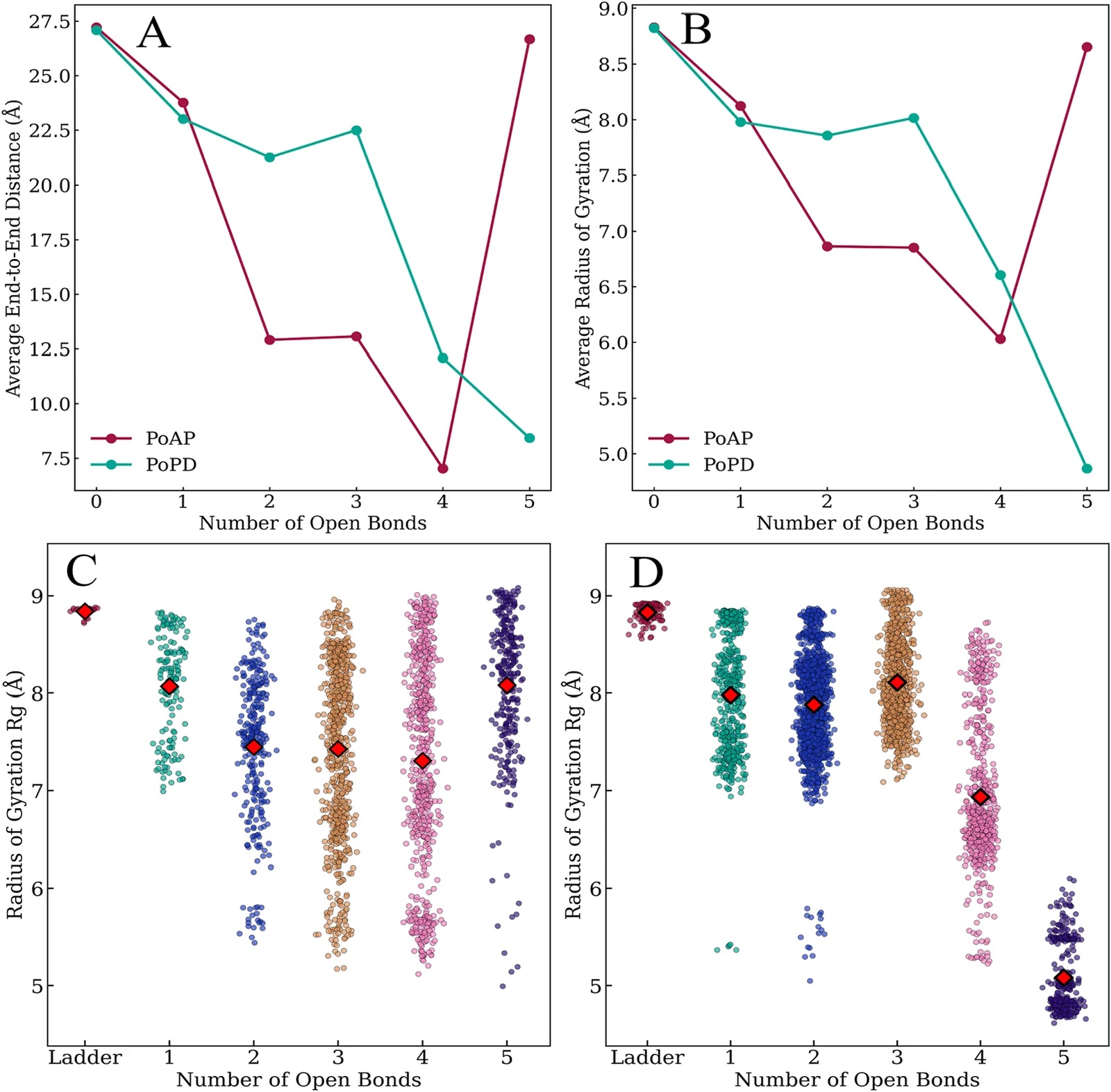
(A) Average end-to-end distance; (B) radius of gyration (*R*_g_) of conformers of PoAP and PoPD hexamers; (C) *R*_g_ scatter plot of PoAP rotamers; and (D) *R*_g_ scatter plot of PoPD rotamers.

A notable observation is that more PoPD samples were generated within the 6 kcal·mol^–1^ energy window and appeared in the rotamer *R*_g_ scatter plots where PoPD points are more densely populated. However, scatter points for PoAP are dispersed at lower *R*_g_ values and are more widely distributed, indicating that low-energy rotations are more accessible for PoAP when both ladder and open bonds are present. For PoAP, the all-open configuration is relatively rigid, with increased end-to-end distance and *R*_g_ in both conformer and rotamer ensembles. In PoAP, the introduction of ladder bonding promotes folding of adjacent open segments, enhancing flexibility. Conversely, the −NH_2_ group in PoPD is less effective at inducing such folding when adjacent to ladder units.

The different behavior of PoPD likely originates from its amine functional group. The −NH_2_ group in the open segments of PoPD has greater degrees of freedom (two hydrogens) than the corresponding −OH functional group in PoAP, which yields more accessible low-energy conformers and rotamers and a larger sample count within the defined energy window. This also explains the pronounced difference in *R*_g_ and end-to-end distance observed for PoPD in the varying bonding configurations. The −NH_2_ groups of PoPD are not induced by adjacent ladder segments to fold the chain as effectively. However, for both hexamers, the largest numbers of samples occur for mixed configurations containing both ladder and open bonds (PoAP: 680 conformers and 715 rotamers for the “three-open” case; PoPD: 1068 conformers and 1409 rotamers for the “two-open” case), while the fewest samples were generated for the fully ladder configuration.

On the basis of the above considerations, the oAP monomer was selected in this step as the best option for imprinting, as its polymer represented more potential for flexibility, a higher electronegativity of oxygen, and, consequently, potential for stronger template–polymer interactions. These factors influence the formation of the imprinted polymer, which exhibits specific and high binding energies for the template.

### 2.3 Prepolymerization Optimization

The effect of the solvent on the formation of the oAP-MEL complex was considered by the inclusion of implicit solvents. In total, six solvents (water, ethanol, acetone, acetonitrile, DMSO, and chloroform) were involved in the calculation. The solvents with a wide range of polarizability properties were selected for these calculations. We screened the binding energies, solvation free energies, and solvent effects on H-bonds in the earlier complexes shown in Figure 1. The solvation model based on density (SMD) is a universal solvation model that uses the full solute electron density. The solvation process involves evaluation of electrostatic interactions using the conductor-like polarizable continuum model (C-PCM) and cavity-dispersion solvent-structure (CDS) models. The latter is the solvent contribution to the short-range interactions between the solute and solvent molecules.^51^ The summary of the calculation results is shown in Table 3. The obtained results revealed that the complex has a higher affinity in nonpolar chloroform than in polar aprotic solvents. This is when the solvent does not interfere with the polar electrostatic attractions and H-bonds. The binding energy of the complex in water as a solvent was the weakest, and ethanol, followed by water, showed the most undesired solvation effect. Three aprotic solvents, including acetone, acetonitrile, and dimethyl sulfoxide (DMSO), were more favorable than protic ones. A higher dielectric (ε) constant increased the solvation energy, and higher Abraham’s hydrogen bond acidity (Sola) reduced the binding energy by weakening H-bonds. This can be seen in protic solvents with debilitated H-bonds. Among all polar solvents, DMSO was found to be the best candidate with good solubility of the oAP and MEL, the highest Abraham’s hydrogen bond basicity (Solb), and maximum binding energy of the complex. A comparison of two applied functionals showed very close values in SMD solvation energies. The binding energies were on average 1.29 kcal·mol^–1^ lower in r^2^SCAN-3c compared with B3LYP-D4, which is in good agreement with the GMTKN55 benchmark database.^52,53^

**Table 3:**
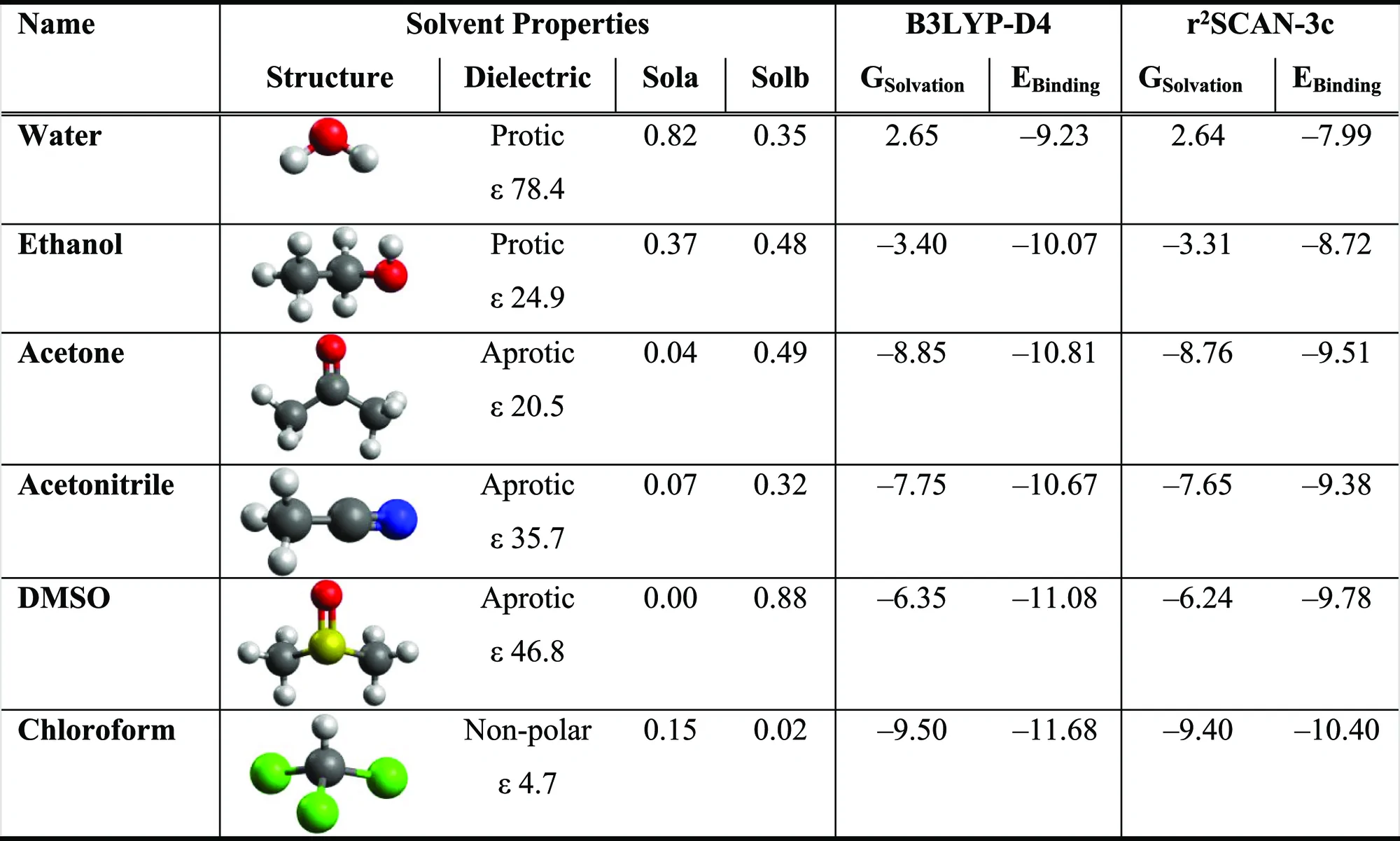
Binding Energy of oAP-MEL Complexes (Ratio: 1:1) in the Solvents in kcal·mol^–1^ ^a^

a The ball-and-stick model of the solvents for inspecting the structure and the corresponding solvent descriptors dielectric (ε) constant, Abraham’s hydrogen bond acidity (Sola), and basicity (Solb) of the solvents are presented.

In molecular imprinting, it is essential to find the optimal template-to-monomer ratio to efficiently fabricate the complementary binding sites. The evaluation of the molar ratio effect was performed using Avogadro software with the MMFF94s force field on 72 complexes, including six independent sets with varying ratios, as presented in Figure 4A, with more details provided in Table S1. The complexes comprised different ratios of oAP-MEL complexes, ranging from 1:1 to 12:1, in which monomers are added around the MEL template in an intuitive sampling method, incrementally placing more monomers and thereafter optimizing. Then, the average number of H-bonds occurring between the MEL template and oAP monomers was obtained. The average number of H-bonds between the oAP monomers and MEL template was maximum at the monomer–template ratio of 10:1 as an indication of optimal conditions. Adding more monomers caused domination of monomer–monomer interactions and reduced the H-bonds between oAP monomers and the MEL template molecules.

**Figure 4:**
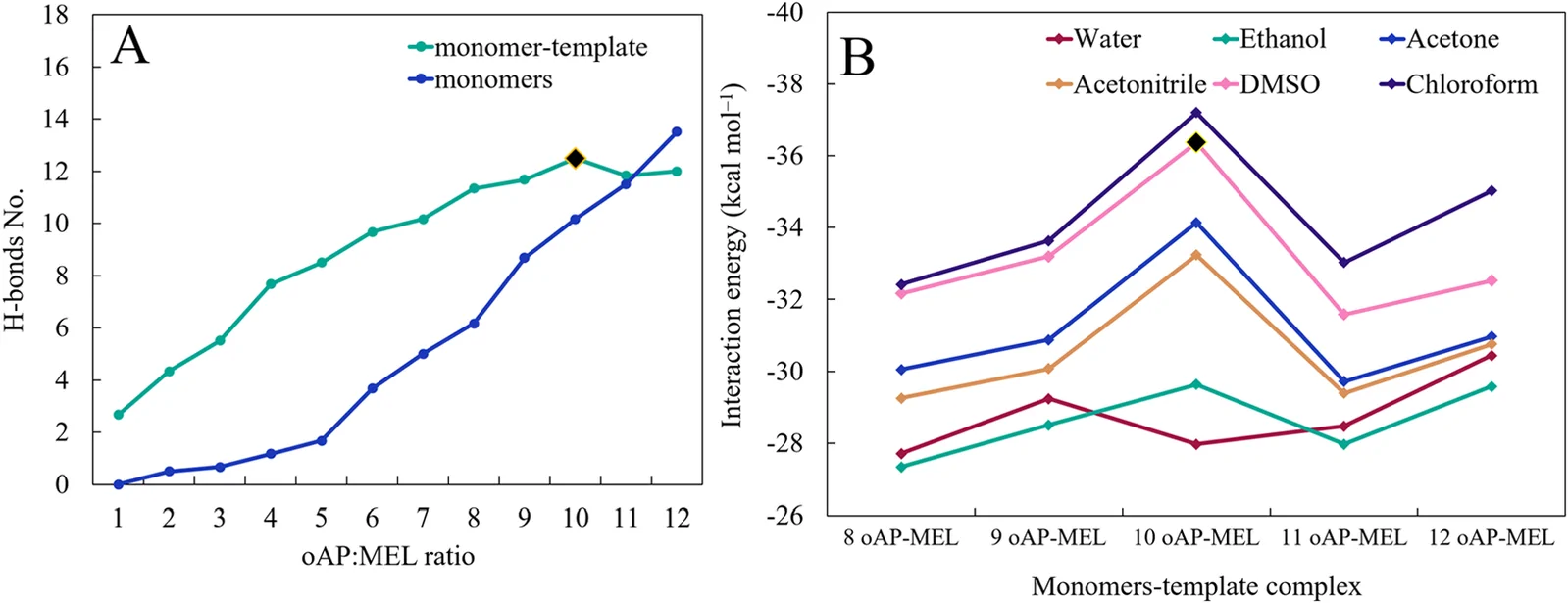
(A) Average number of H-bonds between the oAP monomer(s) and MEL template and H-bonds among monomers in varying ratios in a vacuum. (B) oAP monomer and MEL template interaction energies in the different solvents and at varying ratios from 8:1 to 12:1 oAP:MEL. The highest score was obtained for the complex containing 10 oAP monomers and 1 MEL template molecule in DMSO as a polar solvent.

In the next step, the effects of the number of monomer molecules and solvation were evaluated by calculating the interaction energy of monomers with the template in the solvents. Five complexes containing different monomer–template ratios from 8:1 to 12:1 were used using the solvents mentioned above. All complexes were made by xTB^54^ using the quantum tight-binding GFN2-xTB method.^55^ Then, the energies were calculated with r^2^SCAN-3c.^53^ As Figure 5B demonstrates, the complex 10:1 ratio in all polar nonaqueous solvents had the highest interaction energy, and among them, complexes in DMSO showed the highest scores. Complexes in nonpolar chloroform exhibited the most negative interaction energy due to the minimal solvent interference. However, the increase of the monomer-to-template ratio slightly elevated the interaction energies of monomers with MEL in water. This can be attributed to the poor solubility of the components in the water. The higher number of monomers results in more excluded volume, which prevents short-range hydrogen bond interactions between the solute and solvent. Therefore, an overall increasing trend in the interaction continued in complexes dissolved in water. The obtained 10:1 oAP/MEL ratio and DSMO solvent (marked in Figure 5) were determined to be the optimal choice for MIP formation.

**Figure 5:**
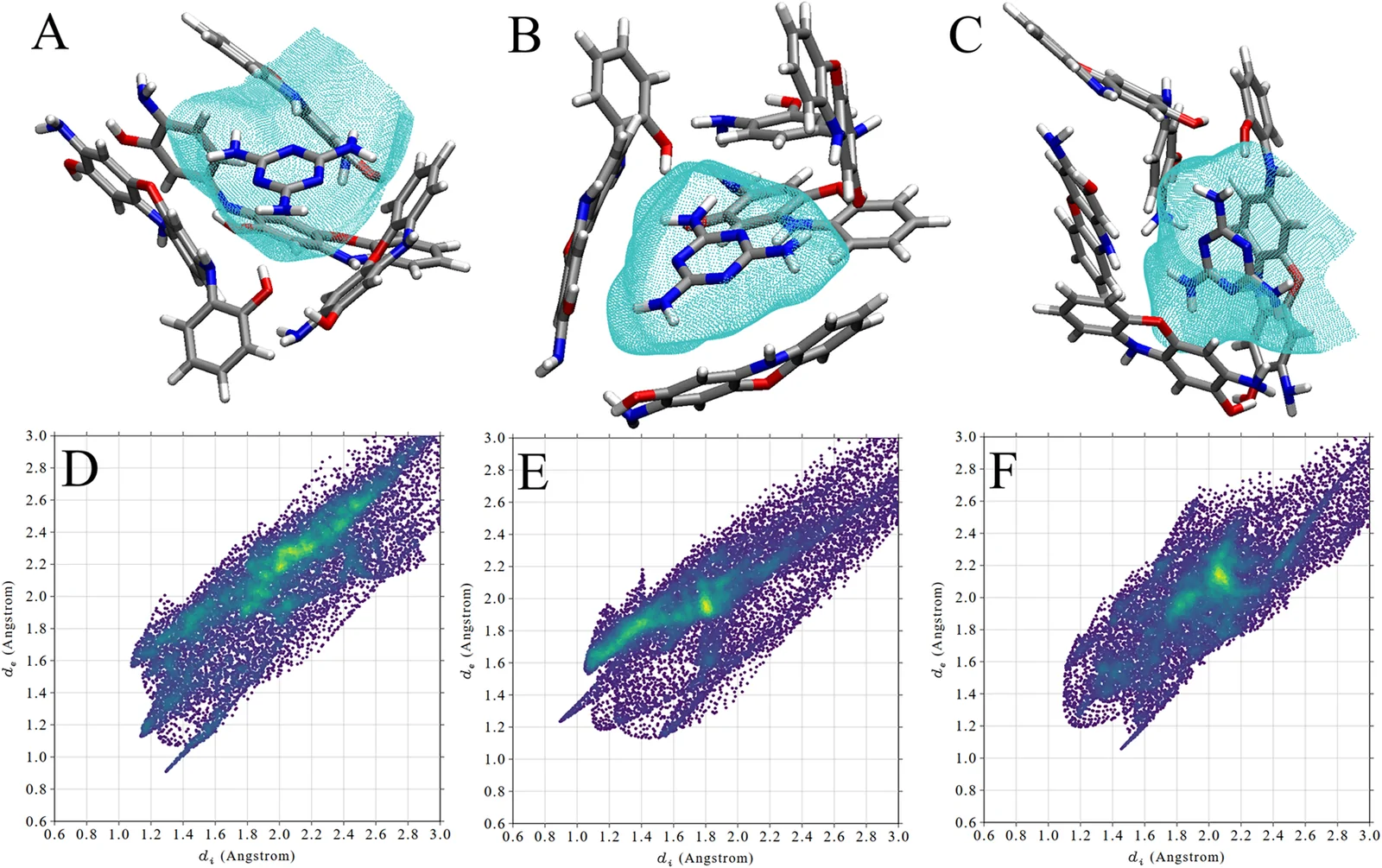
Hirshfeld surface of three binding sites (A, B, and C) and fingerprint plots (D, E, and F) with rebound MEL in three binding sites. The parameters *d*_i_ and *d*_e_ represent the distances from a point on the surface to the nearest nucleus inside and outside the surface, respectively.

### 2.4 MIP Formation and Characterization

The formation of the MIP occurs in two steps: (1) the polymerization of monomers in the presence of the template and (2) subsequent extraction of the template. MIP formation was simulated by considering the monomer:template ratio of 10:1 as optimal in the DMSO solvent (Figure 4). Three complexes with different configurations (comprising two trimers with ladder and open forms and two dimers with ladder configurations) were placed around the template. The aim was to mimic the part of polymer chains that interact with the template molecule, modeling the plausible inclusion of open and ladder configurations. This model forms a cavity-like structure for the template as the binding site. Therefore, geometry optimization (r^2^SCAN-3c) was applied to build the complexes around the MEL template. Neutral forms of molecules with a net charge of zero (pH ≈ 6–8) were used to model complex systems. Then, MEL templates were removed, and hydrogens were relaxed during another geometry optimization. The applied constraints were due to the cavity models, which lack polymeric entanglements and robust polymer network interactions. Therefore, three discrete binding sites (cavities) were made and named the MIP, which are inherently simplified by ignoring segmental moves of the polymer. We assume that no new bond formation/breaking happens during or after the template removal. It should be noted that this simulation approach does not consider the effects of other binding sites and segmental movements. The limited samples for this step are present, aiming at producing unlike binding sites and evaluating their corresponding properties. The coordinates and restrained electrostatic potential (RESP) charges of optimized structures are presented in Table S2.

#### 2.4.1 Hirshfeld Surface Analysis

The imprinting process generates heterogeneous binding sites whose geometries depend on the conformations and configurations adopted by the polymer chains around the template during complexation. This part of the study aims to model the MIP (with a representative of three discrete binding sites) to explore the structural features of these sites and the underlying imprinting mechanism. The results of Hirshfeld surface analysis of imprinted binding sites are shown in Figure 5. The Hirshfeld surface analysis was performed to depict the binding site surface, shape, and size in contact with the MEL template. The mesh surface in Figure 5A–5C illustrates how the atoms of the MEL template are in touch with the binding sites 1, 2, and 3, respectively. The surface analysis measured 346.1, 207.7, and 268.7 Å^3^ volumes of the specified cavities. However, overall surface areas of 174.9, 178.8, and 162.5 Å^2^ were obtained for each binding site, respectively. The differences in volume and surface area between complexes indicate that the imprinting properties varied between the complexes. Binding site 1 (Figure 5A,5D) was formed with a surface-imprinted template. The second complex (Figures 5B,E), with the highest surface area and a significantly lower volume, has the most compact binding site with the template, located deeper within the polymeric network. In the third complex (Figure 5C,5F), the MEL template (re)­bound sidewise. Therefore, the least surface area was obtained in this case. This shows the most compact and largest contact surface complex with the expected highest binding energies for binding site 2. Surface areas of element pairs between fragments are listed in Table S3. The surface area and percentage of the area of element pairs indicate higher hydrogen–hydrogen (H–H) contacts in binding sites 1 and 3. In contrast, a higher nitrogen–hydrogen (N–H) surface area is found in site 2 with MEL. The contact surface between element pairs H–O (H atoms come from MEL and O atoms come from binding sites) is greater than the contact surface between H–N element pairs for all binding sites. This also reflects the higher contact surface of the template with oxygen atoms (hydroxyl or ether functional) at the binding sites. It confirms that the oxygen atom makes a greater contribution than the nitrogen to the noncovalent interaction (NCI), emphasizing the significance of the open form of PoAP.

In Figures 5D,5E,5F, two-dimensional fingerprints of the corresponding binding sites in contact with MEL are displayed to further characterize the close contacts. It reveals the close hydrogen donor-accepting atoms for H-bond formation (classic and aromatic) between two fragments, the template and the binding sites. H-bond acceptors are the right bottom spikes (spikes with *x*-axis > *y*-axis value, where *d*_i_ > *d*_e_), and the left upper spikes (*d*_i_ < *d*_e_) are H-bond-donating signs. The purple, green, and yellow colors indicate that the point density at the corresponding regions is low, medium, and high, respectively. All complexes have purple points along the diagonal of the pictures. Overall, Figure 6E shows spike points at shorter distances, consistent with more pronounced contacts between the N–H element pairs. High-point density regions are noticeable in *d*_i_ < *d*_e_ due to the amine groups interacting with binding sites 1 and 2. Higher densities near the middle of the third binding site stem from its sideway binding into the site.

**Figure 6:**
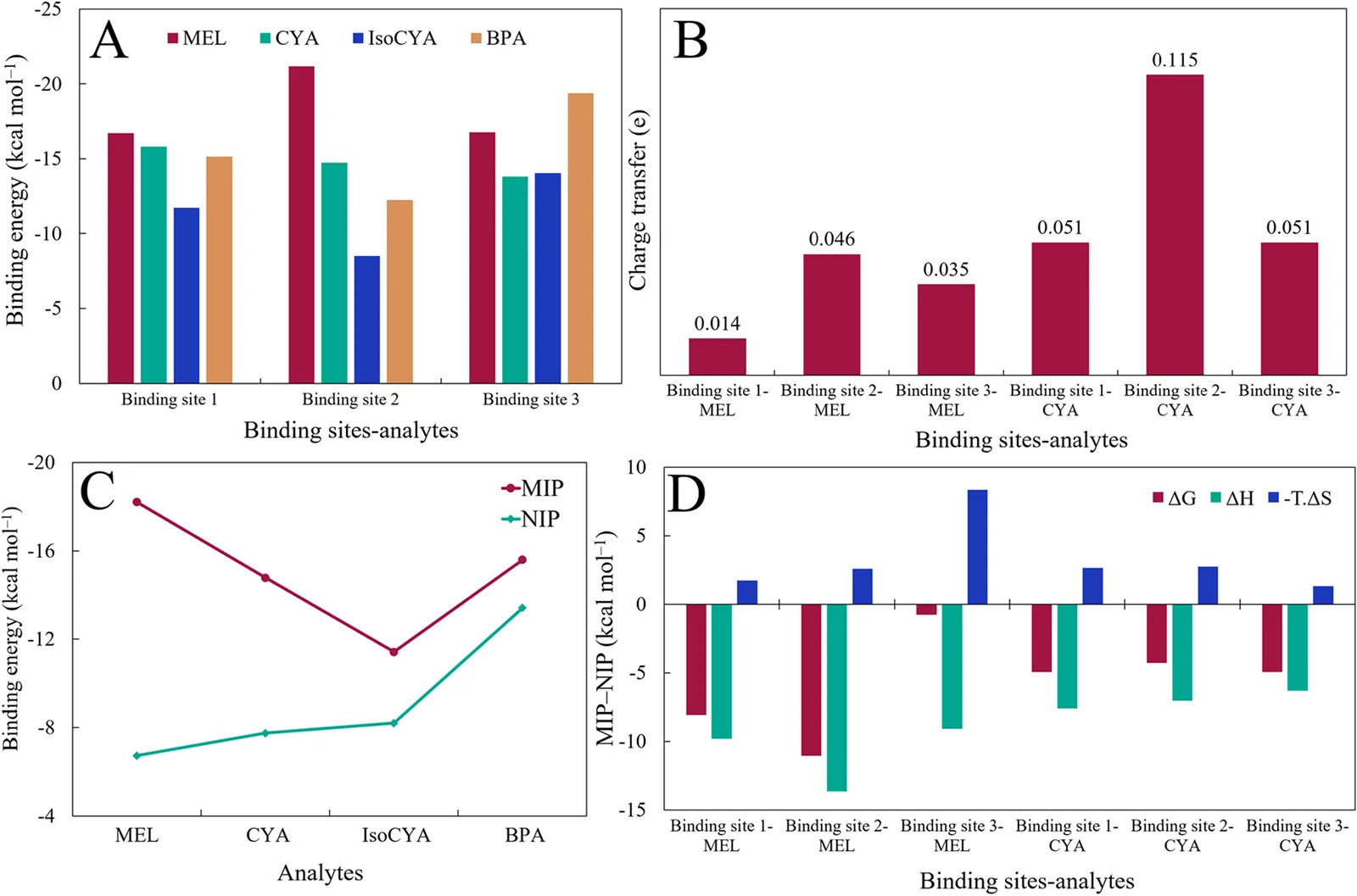
(A) Binding energies of analytes into the three binding sites as the MIP using the r^2^SCAN-3c level of theory in the DMSO solvent. From left: MEL, CYA, IsoCYA, and BPA. (B) Calculated electronic charge transfer is based on the RESP charges of the complex systems. (C) Averaged binding energies (kcal·mol^–1^) of analytes in binding sites, as the MIP and the corresponding PoAP-NIP introduced as the imprinting factor. (D) Free Gibbs energy, enthalpy, and entropy difference between the modeled binding sites and NIP.

#### 2.4.2 Binding Site Analysis

The template was placed inside the cavities, and geometry optimization was performed. Heavy atoms of the binding site were constrained to simulate a firm cavity that delivers reliable, steady interactions. Binding energies were calculated using eq 1 and are shown in Figure 6A. Evaluating the specific binding energies of binding sites and their correlations with interfacial properties can elucidate the underlying imprinting properties. Three binding sites were analyzed with four molecules, which are presented in Figure S1, with MEL as the template and the primary target, as well as CYA (1,3,5-triazine-2,4,6-triol tautomer), isocyanuric acid (IsoCYA; trione tautomer), and bisphenol A (BPA; 4,4′-isopropylidenediphenol). BPA is widely used to make plastics and epoxy resins, serving as one of the primary materials in food containers and other applications.

The acquired binding energies indicate that binding site 2 forms the strongest complex with a binding energy of −21.16 kcal·mol^–1^ for MEL, consistent with its compact geometry and larger interfacial contact area identified in the Hirshfeld analysis. The binding sites demonstrated heterogeneous affinities. Site 1 showed comparable or slightly higher affinity for CYA than for MEL, while site 3 exhibited the strongest interaction with BPA (−19.38 kcal·mol^–1^). These variations suggest that the MIP may have limited discrimination capability when multiple analytes coexist, as different sites may preferentially bind different molecules. The relative energies of binding sites were 3.26 kcal·mol^–1^, 8.32 kcal·mol^–1^, and 0.00 kcal·mol ^–1^, confirming the strongest NCI in binding site 3 and the weakest in binding site 2. The obtained results point to a heterogeneity issue of the cavities and the difficulty of imprinting such a small molecule. The heterogeneity cannot be removed, and each binding site has its unique fingerprint. However, it can be mitigated by a slow polymerization process, which promotes greater polymer relaxation and self-assembly. Likewise, rapid electropolymerization is expected to yield more heterogeneous sites. This suggests that mild polymerization conditions facilitate morphology control, boosting the imprinting efficiency.^56^ Several parameters have been investigated for rational control of electropolymerization for conjugated polymers.^57^

RESP charges are shown in Figure 6B to assess net charge redistribution and polarization upon complexation between each binding site and the targets. The values of charges revealed that the electron charge transfer direction is from the MIP to the MEL and CYA. These charge modulations reflect the sensitivity of the imprinted cavities to the guest orientation, position, and specific conformational arrangement inside the PoAP matrix. Therefore, distinguished recognition behavior is expected for each target. The magnitude of this transfer is generally higher for CYA than for MEL across the sites. This enhanced charge redistribution is consistent with the stronger hydrogen bonding capability of CYA, which possesses three hydroxyl (−OH) groups in its triol tautomer. These −OH groups enable CYA to act as both an effective H-bond acceptor and donor, facilitating stronger noncovalent contacts with the PoAP matrix. Site 2 consistently showed the highest charge transfer value, in line with its more enclosed geometry and greater interfacial area. In contrast, site 1 (surface-exposed) displayed the smallest charge-transfer value for MEL. It is noteworthy that the Hirshfeld fingerprint plots exhibited higher point density in the *d*_i_ < *d*_e_ region, indicating that the PoAP matrix functions primarily as the H-bond donor. This donor–acceptor interaction promotes greater electron density shift from the binding site toward CYA, resulting in larger net negative charge acquisition by the guest. The effect is most noticeable in the more compact binding site 2, where the extended interfacial contact further amplifies this charge modulation. In contrast, MEL, lacking oxygen-based functional groups, exhibits comparatively weaker charge transfer. These observations highlight how structural differences between the MEL and CYA can lead to distinct electronic responses within the imprinted cavities.

#### 2.4.3 Comparison of the MIP and NIP

The NIP is created to be compared with the corresponding MIP as a reference. The fabrication process is identical to that of the MIP, but the template is absent during preparation. This creates a similar polymer film to act as a receptor but lacks a specific binding site. Therefore, in the present work to model an NIP, the same molecular system (2 trimers + 2 dimers) was considered for geometry optimization without the presence of the MEL template, as shown in Figure S3. Then, the heavy atoms of the optimized NIP were constrained, and analytes were bound to the NIP surface via geometry optimization. The binding energy was calculated and compared with the averaged rebinding energies of the analytes into the binding sites, as shown in Figure 6C. This energy ratio was calculated as an approximate measure of the imprinting factor (IF). The average binding energies of the selected molecules in the formed MIP decrease in the order MEL > BPA > CYA > IsoCYA. On the other hand, the IF is highest for MEL (IF = 2.71) and then CYA (IF = 1.91), while it is lowest in the case of BPA (IF = 1.16), indicating minimal improved binding energy. This shows that the enhanced affinity of the studied MIP is highest for MEL, as expected. Similarly, the IF value of the MIP for CYA is quite substantial. The reason behind it is that these two molecules, MEL and CYA, provide a similar molecular conformation space. This results in a similar NCI model within the complex. On the other hand, the BPA shows generally quite strong affinity, albeit the binding of this molecule is mainly nonspecific through the π–π stacking, as shown later in the NCI analysis. Therefore, using MEL as the template to imprint PoAP can also provide an MIP-based sensor for CYA detection. Moreover, IsoCYA showed lower IF values of 1.39. The analysis of binding affinities effectively imprinted MEL for a robust sensor with improved affinities. However, the obtained results indicate that selectivity toward MEL and CYA in the *in silico* design samples in the presence of BPA or similar molecules may be an issue. This issue is linked with such small analytes, including MEL, whereas larger molecules, including BPA, can make more interactions. Larger molecules can engage in multiple nonspecific interactions within the porous PoAP matrix, partially benefiting from the accessible pores created during imprinting, even without precise shape complementarity. Despite this, the *in silico* evaluation was limited to two analogs (BPA and IsoCYA) for computational efficiency; the results highlight the challenge of heterogeneity and cross-reactivity inherent to MIPs, particularly for small templates, including MEL. The PoAP-MIP created a porous structure that increases surface accessibility for all evaluated molecules, with a greater increase for the template.

Thermodynamic parameters (Δ*G*, Δ*H*, and −*T*Δ*S*) were calculated to evaluate the imprinting efficiency and the driving forces for analyte recognition. These parameters were obtained for the binding of MEL and CYA to both the binding sites as MIP and NIP models. The calculations were performed on optimized structures in the DMSO implicit model and are presented in Figure 6D. Binding of the analytes is accompanied by a conformational entropy penalty, as the formation of the host–guest complex restricts the degrees of freedom of both the polymer chains and the guest molecules, thereby reducing flexibility. Comparison between the MIP and NIP reveals that imprinting enhances the enthalpic contribution (more negative Δ*H*), primarily due to improved and more extended NCI within the preorganized cavities. Although the entropy penalty (−*T*Δ*S*) is larger in the MIP than in the NIP, reflecting greater rigidification and loss of conformational freedom upon binding, the overall free energy change (Δ*G*) becomes more favorable (more negative) in the MIP. This indicates that the enthalpic gains outweigh the increased entropic cost, resulting in enhanced binding affinity. Site-specific differences are evident. For MEL, binding site 3 exhibits the largest entropy penalty (most unfavorable −*T*Δ*S* contribution), which dominantly offsets the free energy benefit. In contrast, CYA binding to the same site shows a distinctly smaller entropy penalty, likely arising from differences in conformational accommodation and orientation within the cavity. The most favorable enthalpic contribution for MEL occurs in binding site 2, consistent with previous analyses. These observations underscore the dominant role of conformational entropy in modulating the overall thermodynamics and highlight how the imprinting process creates cavities with distinct energetic signatures for different guests. Overall, while imprinting increases the entropic penalty due to greater rigidification than the more flexible NIP, the substantial improvement in enthalpy makes the process thermodynamically advantageous for recognition.

#### 2.4.4 NCI Analysis

NCI analysis was performed for the qualitative study of inter- and intramolecular NCIs to qualitatively visualize and characterize the inter- and intramolecular interactions within the binding sites. In this analysis, sign­(λ_2_)­ρ is the sign of the second largest eigenvalue of the electron density Hessian matrix (λ_2_) in electron density (ρ). This function, together with the reduced density gradient (RDG), is used to study weak interactions. Figures 7A–7F display the spikes, which represent attractive H-bonds, in the negative value of sign­(λ_2_)­ρ, where ρ has positive values and λ_2_ has negative values. The H-bond spikes are in the blue-green (weaker) and blue regions (stronger). Van der Waals (vdW) interactions, such as dipole–dipole, dipole-induced dipole, and London dispersion interactions, are light green, and spikes appear in the middle of the *x*-axis. These interactions have both λ_2_ and ρ with small values near zero. The dominance of the polar interactions comes from extensive hydrophobic π–π and H-bond π-donor electrostatic interactions. Red spikes in the positive region of sign­(λ_2_)­ρ represent the repulsive forces, mainly stemming from steric hindrance at the center of the rings and intramolecular NCI between adjacent functional groups. In the blue regions, more spikes are observed for the C and F parts of Figure 7, representing the binding site 3 complexed with MEL and CYA. This mainly comes from stronger H-bonds formed between PoAP dimers and trimers of binding site 3.

**Figure 7:**
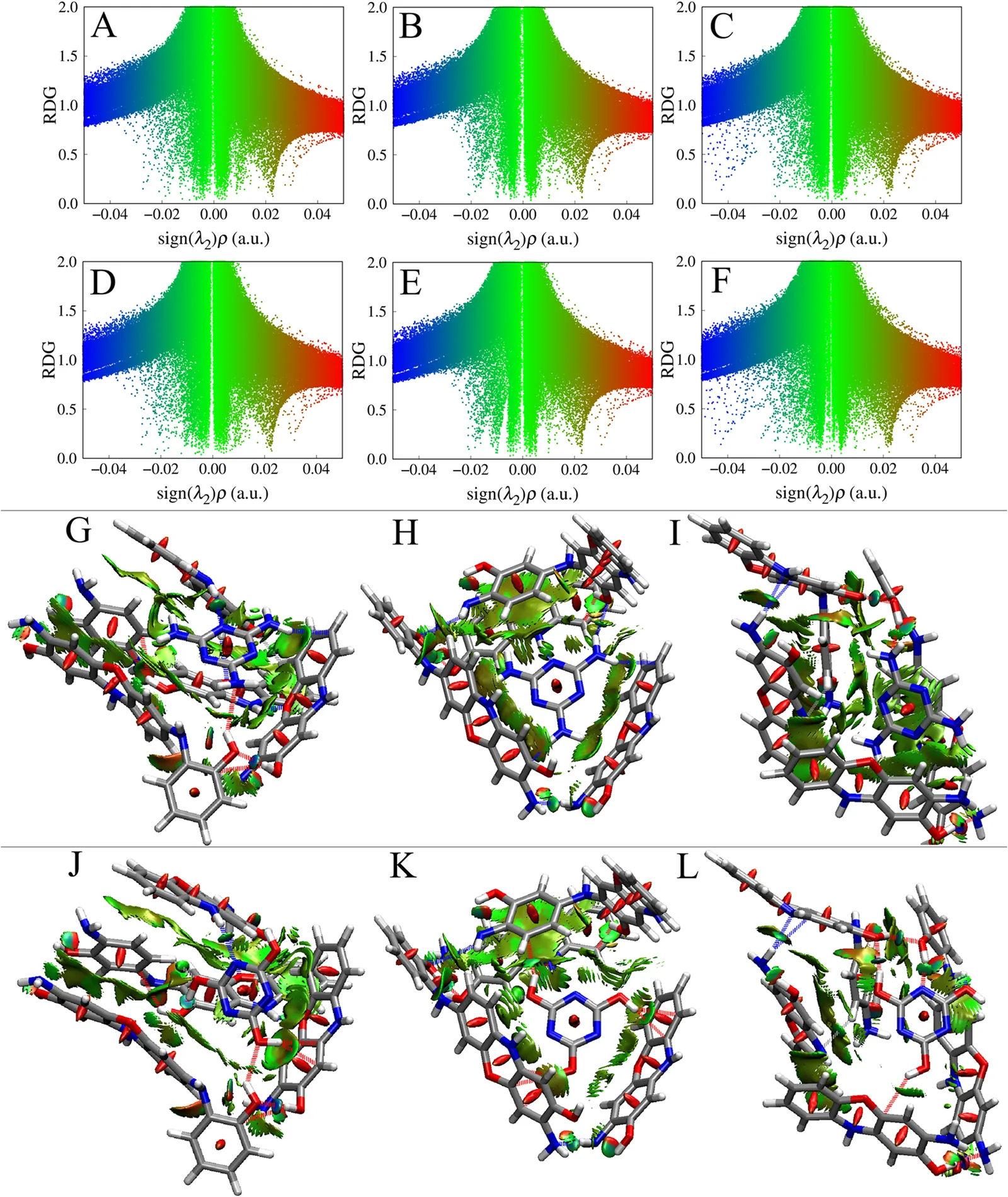
RDG spikes of (A–C) MIP-MEL in three binding sites and (D–F) MIP-CYA in three binding sites. RDG isosurface of (G–I) MIP-MEL in three binding sites and (J–L) MIP-CYA in three binding sites. The dashed lines show detected H-bonds, colored by the atom of the H-donor.

RDG isosurface addresses the interaction regions, providing a spatial visualization that corresponds to the displayed RDG spikes. The results are shown in Figures 7G–7L, using the same color scale, and dashed lines indicate the detected conventional and carbon H-bonds as determined by VMD software. In binding site 2, a more extended green isosurface is observed for MEL compared to CYA, suggesting a larger region of attractive polar and vdW interactions. Conversely, H-bonds are stronger (bluer) for the CYA and IsoCYA complexes with the MIP. A feature across the models is the presence of an extensive green isosurface between aromatic rings, highlighting the contribution of π–π stacking (T-shaped and parallel-displaced) and π-donor H-bond interactions to the stability of the polymeric network. Also, the end of chains and open configuration segments forge strong H-bonds with other chains. The aromatic interactions, together with H-bonds, appear to sustain the overall structure. The isosurface for IsoCYA and BPA bound into the binding sites is presented in Figure S2. IsoCYA formed strong H-bonds in the complexes, whereas the polar interactions (green isosurface) were noticeably reduced. This effect can possibly be used to reduce binding of IsoCYA by introducing for MIP synthesis solvents known for hydrogen bond disruption, which should affect IsoCYA more than MEL binding. A substantial green isosurface can be seen for BPA in binding sites. The imprinted PoAP provides a larger surface area for BPA to interact with its rings. This caused elevated aromatic interactions of PoAP with two phenols of BPA that make several π–π T-shaped and stacked and also π–donor H-bond interactions. Two alkyl groups formed hydrophobic π–alkyl interactions.

Overall, the computational results demonstrate that the designed PoAP-based MIP is well-suited for the imprinting of MEL, exhibiting an enhanced recognition capability toward both MEL and CYA. MEL can effectively serve as a dummy template for the simultaneous detection of the two compounds. However, the presence of BPA, IsoCYA, or similar analogues is likely to cause significant cross-reactivity and interference. The stronger binding affinity of BPA toward PoAP (in both the MIP and NIP) is primarily attributed to its hydrophobic character and extensive nonspecific interactions as a larger molecule. The study highlights the detrimental impact of binding site heterogeneity on selectivity and underscores the importance of developing more structurally controlled imprinting strategies to achieve a higher specificity in the final MIP material. It also demonstrates that the imprinted binding sites are highly sensitive to the conformational arrangement of the polymer and functional groups of the guests, which play a critical role in achieving optimal binding affinity and inducing charge transfer when imprinting small target molecules.

## 3 Conclusions

This article presents a comprehensive molecular modeling and analysis of the step-by-step process of MIP development for MEL and CYA molecule recognition. The calculations enabled the identification of the best monomer and demonstrated the influence of the formed polymer structure on MIP formation. The evaluation of the flexibility and electrochemical properties of oligomers revealed that excessive protonation in the oxidized state caused drastic conformational changes, thereby limiting the possibility of analyte binding in the final material. Therefore, pH-adjusted models, pH ≈ 6–8, were considered to have the system in the neutral and reduced states. The optimal stoichiometry of the prepolymerization complex, as well as the optimal solvent, has also been determined, clearly showing the optimal conditions for MIP preparation. In the final stage, three representative imprinted cavities were constructed, and their interactions with selected analytes were examined in detail. Variations in the cavity structure significantly influenced the binding process, emphasizing the importance of precise control over homogeneous cavity formation. A detailed Hirshfeld surface, RESP charges, and NCI analysis of the analyte binding highlighted the importance of polar and π–π interactions in the MEL and CYA binding within the formed cavities. Thermodynamic evaluations revealed more favorable enthalpic contributions in the MIP, despite a higher entropic penalty compared to the NIP. The results indicate that a PoAP-based MIP may provide a modified polymeric surface for recognizing MEL and CYA targets with improved sensitivity and selectivity.

## 4 Methods

### 4.1 Software and Computational Details

Molecular structures were created using the Merck molecular force field “MMFF94s” using Avogadro 1.2.0 software.^58^ The hybrid (with 0.2 fraction of exact Hartree–Fock exchange) generalized gradient approximation (GGA) functional B3LYP with the def2-TZVP basis set^59^ was used for evaluation of electrochemical properties. The hybrid functional is required to reduce the lack of electron correlation in DFT. The composite meta-GGA DFT method r^2^SCAN-3c^53^ with an auto-auxiliary basis set was used for the interaction studies. All the calculations were performed using ORCA 6 software.^60^ To account for dispersion effects, the D4 atom-pairwise correction based on partial charges was applied, a practice consistent with related computational studies.^61^ This correction enables a more accurate study of noncovalent interactions. Basis set superposition error (BSSE) refers to a computational artifact caused by basis set incompleteness, which results in an artificial overestimation of binding energies. Therefore, geometrical counterpoise correction (gCP) was applied to treat the overbinding effects arising from the BSSE.^62^ A tight SCF iteration was employed, which includes 1 × 10^–8^ energy tolerances between two cycles. The threshold for RMS density changes is 5 × 10^–9^, and the maximum density change was 1 × 10^–7^. The same level of theory was employed for frequency calculations to obtain the thermochemistry of the molecular system at 298.15 K and 1 atm. CREST 3.0 software was used to generate conformers and rotamers of PoAP and PoPD hexamers at the GFN2-xTB level of theory within a 6 kcal mol energy window.^63^ The conformer and rotamer searches are performed using the RMSD collective variable applied in the parallel metadynamics simulations (298.15 K and 1 atm). The metadynamics lengths are automatically set by software based on the flexibility measure. The multilevel ensemble optimization method with tight thresholds and the shake algorithm were imposed on all bonds to optimize the obtained structures in the produced trajectories. The atomic simulation environment was used to analyze the obtained structures.^64^ VMD 1.9.4 software^65^ was used to probe the hydrogen bonds (H-bonds), and IboView v20150427 software was used to produce frontier molecular orbitals.^66^ The H-bonds were detected by VMD using 3.5 Å and 60° cutoff tolerance.^67^ Jmol 16.2.17 was used to produce the MEP,^68^ while Multiwfn 3.8 ^69^ was used to make the Hirshfeld surface and NCI analyses.

### 4.2 Equations and Mathematical Relations

The binding and interaction energies were calculated by using eq 1

1 $$E_{\mathrm{Binding}}=E_{\mathrm{Complex}}-\left(E_{\mathrm{Template}}+E_{\mathrm{Receptor}}\right)$$

where *E* corresponds to the energy of each component. The same method was used to calculate the Free Gibbs energies, enthalpy, and entropy of the complexes. Equations 2–11 were used to characterize oligomers.^70–74^ To this end, HOMO and LUMO energies were obtained in units of eV. Then, using Koopmans’ theorem, energy gap (*E*_g_), ionization potential (IP), and electron affinity (EA) were derived by using eqs 2–4, respectively

2 $$E_{g}=E_{LUMO}-E_{HOMO}$$

3 $$IP=-E_{HOMO}$$

4 $$\mathrm{EA}=-E_{\mathrm{LUMO}}$$

Chemical potential (μ) and Mulliken electronegativity (χ) were calculated from eqs 5 and 6, respectively.

5 $$\mu ={\left(\frac{\partial E}{\partial N}\right)}_{\upsilon \left(r\right)}=-\frac{\mathrm{IP}+\mathrm{EA}}{2}$$

6 $$\chi =-\mu =\frac{\mathrm{IP}+\mathrm{EA}}{2}$$

Equations 7 and 8 were used to obtain “chemical hardness” (η) and softness (*s*), respectively.

7 $$\eta ={\left(\frac{\partial^{2}E}{\partial N^{2}}\right)}_{\upsilon \left(r\right)}={\left(\frac{\partial \mu }{\partial N}\right)}_{\upsilon \left(r\right)}=\frac{\mathrm{IP}-\mathrm{EA}}{2}$$

8 $$s=\frac{1}{\eta }$$

The global electrophilicity (ω) index, nucleophilicity (υ) index, and maximum charge transfer (Δ*N*_MAX_) were obtained using eqs 9, 10, and 11, respectively.

9 $$\omega =\frac{\mu^{2}}{2\eta }$$

10 $$\upsilon =\frac{1}{\omega }$$

11 $$\Delta N_{\max}=\frac{-\mu }{\eta }$$

The Hirshfeld surface analysis was derived from eq 12.^75^ This method is based on Hirshfeld atomic weights, which provide evidence of interfragment surfaces and reveal weak interactions.

12 $$d_{\mathrm{norm}}=\frac{d_{i}-r_{i}^{\mathrm{vdW}}}{r_{i}^{\mathrm{vdW}}}+\frac{d_{e}-r_{e}^{\mathrm{vdW}}}{r_{e}^{\mathrm{vdW}}}$$

where *d*_norm_ is the normalized contact distance, *d*_i_ and *d*_e_ are the distances from a point on the surface to the nearest nucleus inside and outside the surface, respectively, and *r* ^vdW^ is the van der Waals (vdW) radius of the corresponding two atoms.

The RDG is derived from the electron density ρ­(*r*) and its first derivative delta ρ­(*r*) by eq 13

13 $$\mathrm{RDG}\left(r\right)=\frac{1}{2{\left(3\pi^{2}\right)}^{1/3}}\;\frac{\left|\nabla \rho \left(r\right)\right|}{{\rho_{\left(r\right)}}^{4/3}}$$

RDG is a deviation from a homogeneous electron distribution and is dimensionless. RDG has large positive values at large distances (*r*) from the molecule, where the ρ value is approaching zero, and the RDG of nearby atoms is small.^76^

## Supplementary Material



## Notes

H-bond numbers in the prepolymerization complexes, extended *xyz* files for optimized binding site–MEL, contact area between different pairwise elements of binding site–MEL, representation of optimized analytes’ structures, NCI analysis of MIP-IsoCYA and MIP-BPA complexes, and NIP complexed with MEL, CYA, IsoCYA, and BPA (PDF)
