# Supplementary material for: In Silico Design of Molecularly Imprinted Poly(o-aminophenol) for Melamine and Cyanuric Acid Detection and Extraction
Source: ACS Meas Sci Au. 2026 Jul 6;6(4):1285–300. doi: 10.1021/acsmeasuresciau.6c00116 (PMC13495723; doi:10.1021/acsmeasuresciau.6c00116)
Supplement: Supplementary file 1 [file tg6c00116_si_001.pdf]

Supplementary material:

In Silico Design of Molecularly Imprinted Poly(*o*-aminophenol) for Melamine and Cyanuric Acid Detection and Extraction

Enayat Mohsenzadeh [1], Vilma Ratautaite [1, *], Agne Ramanaviciute [2, 3], Krzysztof R. Noworyta [4], Arunas Ramanavicius [1, 2] *

[1] Department of Nanotechnology, State Research Institute Center for Physical Sciences and Technology (FTMC), Sauletekio Ave. 3, LT-10257 Vilnius, Lithuania; enayat.mohsenzadeh@ftmc.lt; vilma.ratautaite@ftmc.lt; arunas.ramanavicius@chf.vu.lt.

[2] Department of Physical Chemistry, Institute of Chemistry, Faculty of Chemistry and Geosciences, Vilnius University (VU), Naugarduko Str. 24, LT-03225 Vilnius, Lithuania;

[3] Department of Physics, University of Cambridge, Cavendish Laboratory, JJ Thomson Avenue, Cambridge, CB3 0HE, United Kingdom; ar2301@cam.ac.uk.

[4] Institute of Physical Chemistry, Polish Academy of Sciences. Kasprzak 44/52, 01-224 Warsaw, Poland. knoworyta@ichf.edu.pl.

* Corresponding authors: Dr Vilma Ratautaite (vilma.ratautaite@ftmc.lt); Prof. habil. Dr Arunas Ramanavicius (arunas.ramanavicius@chf.vu.lt)

PhD student Enayat Mohsenzadeh https://orcid.org/0000-0002-6598-7622
Dr. Vilma Ratautaite https://orcid.org/0000-0002-9931-4397
PhD student Agne Ramanaviciute https://orcid.org/0009-0005-3997-6160
Prof. habil. Dr. Krzysztof Noworyta https://orcid.org/0000-0002-9883-1288
Prof. habil. Dr. Arunas Ramanavicius https://orcid.org/0000-0002-0885-3556

Pre-polymerization optimization

The study of molar ratio optimization involved placing an increasing number of oAP monomers around the melamine template. This process continued until the maximal number of H-bonds was formed between monomers and template molecules. Six complex sets were considered to gain a probabilistic approach when optimized by the MMFF94s force field. According to the data in Table S3, the maximal number of monomer-template H-bonds should be formed in the polymerization solution containing monomer and template concentrations at a 10:1 molar ratio.

Table S1. Molar ratio optimization based on averaged H-bonds formed between oAP monomers and melamine template. Monomer-monomer and monomer(s)-template columns report the number of H-bonds found among the oAP monomers and oAP monomers with MEL template, respectively.

**Ratio of monomer to template**	**Complex set 1**		**Complex set 2**		**Complex set 3**		**Complex set 4**		**Complex set 5**		**Complex set 6**		**Averages**	
	Monomers -monomer	Monomers -template	Monomers -monomer	Monomers -template	Monomers -monomer	Monomers -template	Monomers -monomer	Monomers -template	Monomers -monomer	Monomers -template	Monomers -monomer	Monomers -template	Hbonds monomer-monomer	Hbonds Monomers -template
**1 *o*AP-Mel**	0	3	0	3	0	2	0	2	0	4	0	2	0.00	2.67
**2 *o*AP-Mel**	0	6	2	3	0	5	0	5	0	5	1	2	0.50	4.33
**3 oAP-Mel**	0	9	2	5	1	6	0	5	0	5	1	3	0.67	5.50
**4 oAP-Mel**	0	11	2	8	2	7	0	7	1	9	2	4	1.17	7.67
**5 oAP-Mel**	1	11	2	9	3	9	1	6	1	9	2	7	1.67	8.50
**6 oAP-Mel**	2	11	5	10	6	9	2	7	4	11	3	10	3.67	9.67
**7oAP-Mel**	5	10	4	10	8	9	4	8	4	11	5	13	5.00	10.17
**8 oAP-Mel**	7	9	8	11	7	10	4	11	4	13	7	14	6.17	11.33
**9 oAP-Mel**	13	12	9	12	9	11	4	10	9	10	8	15	8.67	11.67
**10 oAP-Mel**	**13**	**13**	**13**	**13**	**10**	**12**	**7**	**11**	**10**	**12**	**8**	**14**	**10.17**	**12.50**
**11 oAP-Mel**	16	10	14	15	11	12	9	11	12	11	7	12	11.50	11.83
**12 oAP-Mel**	14	10	16	15	14	13	11	11	12	11	14	12	13.50	12.00

MIP formation and characterization

Table S2. The extended xyz files for modeled binding sites-MEL complexes presenting optimized coordinates and obtained RESP charges.

**Binding site 1-MEL**	**Binding site 2-MEL**	**Binding site 3-MEL**
145	145	145
# ORCA CHELPG point charges xyz file	# ORCA CHELPG point charges xyz file	# ORCA CHELPG point charges xyz file
C 1.025600 14.612800 1.967600 -0.37030982	C 2.888900 14.305800 4.316800 -0.31275886	C 7.580000 16.772900 -5.005400 -0.31444574
C 0.812000 14.808400 -0.804800 -0.22259820	C 3.850200 11.815400 5.122400 -0.33332551	C 8.485300 14.635500 -6.551800 -0.34052299
C 1.730600 15.606800 1.282500 -0.16958546	C 2.727300 13.890400 5.641000 -0.18800757	C 8.830100 16.838900 -5.624000 -0.20458890
C 0.214900 13.738800 1.265900 0.38156638	C 3.540500 13.484000 3.413900 0.26548115	C 6.802000 15.637000 -5.150200 0.29621888
C 0.101600 13.815400 -0.130800 0.14784348	C 4.029800 12.228600 3.803300 0.24466318	C 7.243900 14.554800 -5.924300 0.25320645
C 1.617500 15.702300 -0.099600 -0.26233153	C 3.207200 12.647300 6.038500 -0.19616321	C 9.277600 15.772000 -6.396100 -0.18986999
H 2.358801 16.297893 1.835884 0.17377747	H 2.224739 14.541345 6.349871 0.17218823	H 9.443160 17.726486 -5.500970 0.17870604
O -0.500000 12.798800 1.992500 -0.35999507	O 3.735600 13.954000 2.127700 -0.31389958	O 5.594700 15.583500 -4.475900 -0.34577701
N -0.710200 12.901500 -0.791200 -0.57475265	N 4.701000 11.451200 2.861800 -0.53311081	N 6.437400 13.415900 -6.001600 -0.50531697
H 2.158706 16.471730 -0.642417 0.19134024	H 3.083312 12.313944 7.064739 0.17778651	H 10.246357 15.817131 -6.884922 0.17986736
H 0.727211 14.877540 -1.886757 0.17958281	H 4.227317 10.841292 5.424908 0.20990971	H 8.827978 13.797494 -7.154041 0.21032960
H 1.093077 14.517600 3.048011 0.21768820	H 2.521203 15.271178 3.980546 0.20164935	H 7.206367 17.594576 -4.400924 0.20761026
C -1.697500 10.671900 -0.766600 -0.56293406	C 4.880800 10.837600 0.513400 -0.51909425	C 4.135000 12.619500 -6.117200 -0.37021350
C -1.479400 10.636100 1.998800 -0.35732008	C 3.864700 13.349300 -0.168000 -0.41028243	C 3.354500 14.786000 -4.547800 -0.29250487
C -1.140900 11.765200 -0.112000 0.34335491	C 4.538900 11.744000 1.513300 0.22729052	C 5.079500 13.552500 -5.704800 0.12517096
C -2.204100 9.588500 -0.046300 0.28659991	C 4.738600 11.201600 -0.825200 0.26312409	C 2.796100 12.770900 -5.754400 0.23265919
C -2.090500 9.550800 1.352100 0.05547471	C 4.226900 12.451900 -1.178300 0.10669308	C 2.400300 13.844000 -4.946300 -0.04374167
C -1.037000 11.728500 1.281800 0.18068308	C 4.035100 13.005300 1.157200 0.21550304	C 4.673400 14.647000 -4.931900 0.17231443
O -2.863200 8.642500 -0.770500 -0.40839739	O 5.106800 10.305500 -1.783400 -0.46303151	O 1.910000 11.845500 -6.206100 -0.50583580
N -2.697700 8.535300 2.114900 -0.55405387	N 4.059100 12.739600 -2.565700 -0.43577581	N 1.039700 13.955800 -4.556600 -0.35114567
H -1.380661 10.629565 3.081590 0.22666614	H 3.455211 14.322687 -0.425027 0.24237303	H 3.059723 15.623714 -3.921295 0.22481838
H -1.799932 10.678552 -1.848802 0.25396441	H 5.274155 9.858443 0.773630 0.28105017	H 4.435666 11.777707 -6.735368 0.23619758
H -3.016364 7.835355 -0.253380 0.26270935	H 4.981967 10.742959 -2.649305 0.29700952	H 0.997787 12.139984 -6.017068 0.39877206
H -0.629767 12.844153 -1.799151 0.36425286	H 4.892764 10.489246 3.108815 0.40608263	H 6.681514 12.734220 -6.709368 0.40188680
H -0.503614 4.844555 0.448388 0.18237898	H -0.359814 11.471933 -2.647712 0.19512910	H 1.191828 10.500881 -1.543201 0.20534900
H -1.318782 2.628142 2.790620 0.37804691	H -1.746477 12.194522 -4.467256 0.45280457	H -0.384909 10.594611 0.358350 0.45533992
C -1.247800 5.256500 1.125500 -0.12308325	C 0.458800 12.003700 -3.129400 -0.28844874	C 0.684500 11.391300 -1.897300 -0.18794828
H -1.209454 2.683879 1.159775 0.41594757	H -0.963594 12.079416 -5.900697 0.40563832	H -1.801564 10.450343 -0.448744 0.41914058
N -1.773400 2.977700 1.950100 -0.90015344	N -1.017400 12.614200 -5.035000 -1.05613449	N -1.085000 11.116000 -0.161800 -1.06455336
C -1.470500 6.636300 1.144400 -0.52160365	C 1.703300 12.057900 -2.500200 -0.44306568	C 1.231200 12.109700 -2.957700 -0.35295344

C	-1.925800	4.390100	1.970800	0.14141636	C	0.230800	12.622700	-4.353500	0.35908738	C	-0.502700	11.775200	-1.287900	0.19225136
C	-2.405200	7.171800	2.041000	0.38811246	C	2.759500	12.738600	-3.113300	0.33426487	C	0.561300	13.237400	-3.448900	0.20879954
C	-2.886800	4.940000	2.841100	0.37464267	C	1.284600	13.364500	-4.922400	0.23990406	C	-1.142400	12.934300	-1.762800	0.35937516
C	-3.118900	6.303900	2.886200	-0.56754437	C	2.535800	13.405700	-4.324700	-0.52994710	C	-0.634800	13.647400	-2.840900	-0.52301846
O	-3.624100	4.122200	3.648000	-0.54254941	O	1.087800	14.074200	-6.068300	-0.49633336	O	-2.276100	13.379200	-1.150100	-0.57438106
H	-3.876395	6.696047	3.560419	0.25045911	H	3.335466	13.968435	-4.799984	0.26034443	H	-1.154365	14.536652	-3.190178	0.25607148
H	-3.492341	3.213122	3.324867	0.41393361	H	0.139407	14.031915	-6.275868	0.39364288	H	-2.384371	12.818560	-0.358790	0.42638334
H	-3.066477	8.839058	3.005572	0.40601866	H	4.629556	13.506525	-2.903051	0.33965844	H	0.668830	14.895921	-4.616662	0.34835528
C	-5.416200	9.786300	-9.195400	-0.31260118	C	-4.408800	13.691100	-6.871200	-0.35334957	C	-1.957400	13.746000	-6.262200	-0.25486938
C	-6.546200	8.061900	-7.313100	-0.17916449	C	-5.443300	12.292200	-4.695600	-0.25834373	C	-4.126400	12.707500	-4.848200	-0.27135351
C	-6.788700	9.795300	-8.970800	-0.16430322	C	-5.299600	14.313100	-6.002700	-0.17133858	C	-3.004100	14.578400	-5.877800	-0.24394718
C	-4.611100	8.917400	-8.464600	0.30382121	C	-4.027800	12.371300	-6.646700	0.33877177	C	-1.985100	12.399700	-5.916300	0.28318253
C	-5.168500	8.037900	-7.526900	0.11423649	C	-4.541900	11.654700	-5.550300	0.15854998	C	-3.085300	11.858000	-5.227400	0.19699333
C	-7.355300	8.940200	-8.026200	-0.22450627	C	-5.816300	13.614800	-4.912800	-0.22796356	C	-4.081100	14.063300	-5.158200	-0.17598665
H	-7.416911	10.477334	-9.536643	0.17283614	H	-5.587863	15.345471	-6.179622	0.17476174	H	-2.972167	15.632982	-6.136633	0.18717917
O	-3.255700	8.940700	-8.672100	-0.48799941	O	-3.153400	11.795700	-7.520100	-0.53049837	O	-0.925500	11.612400	-6.273800	-0.54586242
N	-4.275200	7.172500	-6.830500	-0.72525708	N	-4.103100	10.316200	-5.381100	-0.65394363	N	-3.065100	10.470700	-4.973300	-0.68826004
H	-8.427390	8.949000	-7.853146	0.17447324	H	-6.518938	14.093199	-4.236730	0.17594742	H	-4.898854	14.711067	-4.855904	0.16548882
H	-6.977954	7.386183	-6.579429	0.16761134	H	-5.854276	11.741869	-3.854321	0.19739138	H	-4.980686	12.295962	-4.318422	0.18827316
H	-4.964175	10.452892	-9.924677	0.21250357	H	-3.990708	14.221109	-7.722625	0.21743621	H	-1.099826	14.134542	-6.803960	0.18239452
H	-2.855739	8.402230	-7.960812	0.37963180	H	-2.895671	10.914405	-7.193231	0.36310213	H	-0.973799	10.777320	-5.769146	0.39529863
C	-4.135200	5.681300	-4.920900	-0.79841657	C	-4.226200	8.304700	-4.046400	-0.60461100	C	-3.900700	8.478700	-3.928900	-0.64736456
C	-4.917200	7.733500	-3.216400	-0.04363077	C	-3.596100	9.720400	-1.743300	-0.33787820	C	-4.122200	9.767200	-1.479600	-0.22763939
C	-4.436500	6.942200	-5.450600	0.74309262	C	-4.023500	9.692600	-4.124900	0.50382315	C	-3.588700	9.844500	-3.839900	0.58027757
C	-4.196400	5.475800	-3.552300	0.62588329	C	-4.129500	7.654600	-2.827600	0.32618011	C	-4.273100	7.778600	-2.799100	0.45011892
C	-4.600700	6.489600	-2.676000	-0.33395451	C	-3.845300	8.353700	-1.647100	0.26522861	C	-4.395300	8.404500	-1.553200	0.06098756
C	-4.822600	7.970200	-4.584200	-0.59839094	C	-3.677800	10.388900	-2.962700	-0.42369809	C	-3.720900	10.487400	-2.604600	-0.50777292
O	-3.811400	4.235200	-3.076500	-0.35976605	O	-4.375700	6.295700	-2.810800	-0.28604800	O	-4.539600	6.428300	-2.934300	-0.34905918
N	-4.628400	6.229800	-1.293400	-0.06212477	N	-3.854100	7.664000	-0.429300	-0.72535603	N	-4.801700	7.631300	-0.452900	-0.60136336
H	-5.052188	8.957378	-4.974535	0.25204442	H	-3.479914	11.455285	-2.995188	0.24695195	H	-3.488653	11.541477	-2.503034	0.26869436
H	-5.220441	8.535894	-2.549401	0.17147301	H	-3.339844	10.274164	-0.843060	0.22028730	H	-4.220535	10.275629	-0.522556	0.20972947
H	-3.830931	4.862444	-5.567964	0.26735570	H	-4.469317	7.728047	-4.935575	0.25020313	H	-3.799474	7.946205	-4.871763	0.24890570
H	-4.034614	6.334236	-7.349533	0.42513255	H	-4.417386	9.685991	-6.111530	0.40673099	H	-3.048369	9.883997	-5.799237	0.41561433
C	-4.720700	4.875000	-0.921500	0.01343115	C	-3.706000	6.272900	-0.477900	0.46902146	C	-4.502100	6.264000	-0.521500	0.30124852
C	-4.315200	2.550800	-1.475500	-0.43382276	C	-3.797500	4.233000	-1.782400	-0.46600594	C	-4.040700	4.334100	-1.913000	-0.49925764
C	-5.195600	4.473700	0.326600	-0.48817678	C	-3.340900	5.520200	0.637000	-0.67667781	C	-4.381500	5.458300	0.610000	-0.60800754

C	-4.758000	2.141100	-0.213300	0.30916831	C	-3.433300	3.468900	-0.667100	0.09502802	C	-3.927000	3.520500	-0.783800	0.15678219
H	-5.373553	0.556849	0.897379	0.42073259	H	-3.304414	1.602338	0.116344	0.30892231	H	-4.083320	1.567277	-0.241384	0.36383461
C	-5.207500	3.128400	0.678500	0.18923979	C	-3.213200	4.136900	0.546000	0.30154119	C	-4.112100	4.098300	0.479200	0.28200368
H	-3.973892	1.813971	-2.198090	0.25039129	H	-3.974703	3.747040	-2.738551	0.26474382	H	-3.914456	3.910825	-2.906382	0.26736601
H	-5.544058	5.224689	1.033550	0.25222041	H	-3.147995	6.022261	1.583606	0.28494414	H	-4.508262	5.895870	1.598703	0.27986794
N	-4.694800	0.802900	0.188700	-0.94972710	N	-3.210500	2.084600	-0.769400	-0.53814342	N	-3.560600	2.155300	-0.884600	-0.67895771
H	-4.729905	0.148604	-0.582467	0.42420624	H	-3.797290	1.642375	-1.467274	0.32338826	H	-3.687688	1.800822	-1.826841	0.36722213
O	-5.662000	2.687800	1.897900	-0.55252288	O	-2.860600	3.358600	1.618300	-0.55809824	O	-4.015200	3.261400	1.558600	-0.55540554
C	-4.294100	3.894300	-1.814100	0.18913024	C	-3.945800	5.606000	-1.680500	0.11431869	C	-4.342000	5.678300	-1.778600	0.24370768
H	-5.237421	6.846122	-0.767513	0.30577445	H	-3.403004	8.131949	0.346316	0.44415280	H	-4.657060	8.061080	0.452335	0.42544465
C	2.327400	12.087600	-2.668700	-0.40476942	C	5.417400	11.576100	-6.469600	-0.28418802	C	1.748800	11.731900	2.237700	-0.28345913
C	3.716400	11.939500	-5.081100	-0.37185714	C	4.659100	9.479800	-4.796400	-0.29388616	C	1.557000	9.026700	1.613900	-0.19453778
C	3.703600	11.845800	-2.665900	-0.16790741	C	6.371200	10.950300	-5.661900	-0.26872860	C	0.934000	10.898200	3.007800	-0.24590986
C	1.665900	12.274300	-3.868400	0.26344109	C	4.097200	11.165100	-6.423300	0.22506795	C	2.453800	11.215700	1.163400	0.29408368
C	2.344600	12.200000	-5.093600	0.28457464	C	3.693800	10.122200	-5.572500	0.25162219	C	2.364700	9.853000	0.829100	0.17761189
C	4.392500	11.776200	-3.872200	-0.13992802	C	5.990900	9.898700	-4.836100	-0.16507193	C	0.845400	9.546600	2.695600	-0.24793046
H	4.221091	11.707892	-1.721644	0.15142530	H	7.404400	11.281992	-5.694576	0.19649353	H	0.379204	11.312020	3.844280	0.19094139
O	0.309600	12.582500	-3.828500	-0.19464593	O	3.181300	11.835300	-7.209800	-0.23822490	O	3.233000	12.093500	0.436200	-0.34375547
N	1.632000	12.408700	-6.268600	-0.64567255	N	2.346800	9.790700	-5.522200	-0.53233843	N	3.031400	9.386000	-0.294100	-0.46951082
H	5.461507	11.583523	-3.883236	0.16298229	H	6.727454	9.391700	-4.219248	0.17033175	H	0.221102	8.884027	3.288128	0.18845108
H	1.773175	12.128619	-1.736560	0.21484371	H	5.690612	12.392024	-7.132941	0.19820566	H	1.841832	12.790592	2.463517	0.19572839
C	-0.531500	12.032100	-7.362400	-0.56507792	C	0.303500	9.565700	-6.850800	-0.50542585	C	4.754200	9.764200	-1.982000	-0.41841899
C	-1.787500	12.224900	-4.889600	-0.44535466	C	1.181800	11.650600	-8.476000	-0.35351129	C	4.931700	12.418200	-1.180400	-0.48068758
C	0.239400	12.254000	-6.224700	0.36604709	C	1.532900	10.168200	-6.592400	0.22164296	C	3.944600	10.220200	-0.943600	0.07896876
C	-1.917200	11.916700	-7.274700	0.36341774	C	-0.482500	10.016600	-7.905200	0.19464832	C	5.662800	10.619000	-2.597700	0.18856084
C	-2.571900	12.014200	-6.035700	0.20804632	C	-0.071300	11.079600	-8.718800	0.31890685	C	5.766100	11.962600	-2.207400	0.34195853
C	-0.413900	12.342600	-4.993300	0.02892486	C	1.967800	11.198500	-7.430900	0.08040481	C	4.040600	11.559300	-0.559800	0.24788647
O	-2.665100	11.742300	-8.424600	-0.55095805	O	-1.689800	9.393200	-8.128900	-0.52955333	O	6.457400	10.104300	-3.590400	-0.53331836
N	-3.961000	11.940500	-5.955800	-0.89059404	N	-0.909700	11.470200	-9.791400	-1.00646636	N	6.764200	12.772900	-2.783100	-0.99147613
H	-2.258083	12.295823	-3.911666	0.28272797	H	1.534714	12.474118	-9.091114	0.21376908	H	4.981511	13.455797	-0.859585	0.24642272
H	-4.309360	11.590031	-5.073221	0.42421059	H	-0.448926	12.136655	-10.400141	0.44365370	H	6.733868	13.724981	-2.438417	0.45696496
H	-4.394780	11.471588	-6.741192	0.38272438	H	-1.774555	11.892971	-9.462144	0.40215151	H	6.739317	12.791708	-3.799852	0.38468737
H	-0.053094	11.948737	-8.335224	0.24675864	H	-0.036028	8.731799	-6.243273	0.28347504	H	4.699984	8.725187	-2.294226	0.27028128
H	-2.779249	10.786381	-8.583020	0.33567558	H	-1.964167	9.620259	-9.035001	0.42014889	H	7.228642	10.682376	-3.697621	0.41584139
H	2.090158	12.129843	-7.126503	0.42735564	H	2.085507	8.972549	-4.985874	0.39955166	H	3.140008	8.385462	-0.400972	0.37826778
H	-0.877205	7.283705	0.504368	0.22319305	H	1.848412	11.557193	-1.548006	0.21649925	H	2.178184	11.797125	-3.388536	0.18975541

C	1.942700	3.964500	2.097300	-0.35198524	C	5.185500	5.301000	-3.210100	-0.33711072	C	5.063700	6.228500	-0.821800	-0.30860115
O	2.248900	6.275300	2.494000	-0.32654428	O	3.679000	3.512400	-2.806800	-0.34693424	O	3.101200	5.038200	-1.412900	-0.34022253
C	2.170200	7.566100	1.985100	0.21556547	C	2.774400	2.964400	-1.902500	0.32517741	C	2.063000	4.233600	-0.956500	0.26150282
C	2.016000	8.600000	2.893800	-0.48232579	C	1.647200	2.340300	-2.409100	-0.59398907	C	1.532200	3.311900	-1.840600	-0.49755714
C	1.995500	9.928900	2.460700	0.39075769	C	0.723600	1.732700	-1.551600	0.42605688	C	0.443900	2.510300	-1.469600	0.35392171
C	2.140600	10.182700	1.089900	0.08131536	C	0.980200	1.759800	-0.171500	0.17838759	C	-0.068000	2.639800	-0.164800	0.25812490
O	2.196900	11.466300	0.616600	-0.33342420	O	0.142700	1.141900	0.715300	-0.51650215	O	-1.054700	1.805100	0.290000	-0.57285928
H	2.313967	12.064277	1.373847	0.32770303	H	-0.489719	0.607150	0.210013	0.41034030	H	-1.906404	1.939328	-0.211715	0.36460277
C	2.244600	9.140200	0.173900	-0.59710908	C	2.093100	2.426400	0.336200	-0.48199397	C	0.481400	3.573200	0.713800	-0.45535302
H	2.348635	9.368842	-0.882633	0.30751558	H	2.261528	2.450793	1.409764	0.27438544	H	0.062568	3.663488	1.713214	0.24150301
H	1.913492	8.369811	3.951434	0.23601876	H	1.476260	2.337753	-3.482680	0.26127513	H	1.957318	3.227562	-2.837929	0.25132794
C	2.269800	7.817100	0.614200	0.34295992	C	3.003500	3.032900	-0.527400	0.14332419	C	1.546000	4.384300	0.329900	0.02942035
N	2.437700	6.749200	-0.273600	-0.71050631	N	4.153200	3.685400	-0.066900	-0.58029300	N	2.085000	5.373700	1.168700	-0.36240228
H	2.151845	6.921281	-1.239766	0.44672445	H	4.185117	3.911158	0.918685	0.41550369	H	1.862899	5.299677	2.153121	0.37157055
C	2.231111	5.458237	0.205823	0.36172810	C	4.796516	4.567055	-0.929742	0.26229769	C	3.343333	5.875440	0.854694	0.16744298
C	2.125448	4.361323	-0.651420	-0.35377877	C	5.704981	5.527292	-0.481135	-0.34599750	C	4.113043	6.593874	1.771695	-0.34110408
H	2.208477	4.524068	-1.723523	0.20242166	H	5.898262	5.610523	0.585938	0.20728135	H	3.733200	6.731512	2.781368	0.20362414
C	1.926000	3.077800	-0.143000	-0.25123517	C	6.364400	6.355600	-1.390400	-0.18619926	C	5.338900	7.139400	1.392900	-0.17864146
H	1.846545	2.239685	-0.829379	0.19387271	H	7.074305	7.090982	-1.022476	0.17447312	H	5.916746	7.705061	2.118185	0.17450511
C	1.836200	2.874700	1.229400	-0.16753213	C	6.109500	6.244900	-2.752700	-0.21413732	C	5.817400	6.960200	0.099300	-0.21390311
H	1.865816	3.835259	3.173493	0.20917256	H	4.962859	5.199646	-4.268744	0.20330945	H	5.414841	6.072436	-1.838077	0.20122417
C	2.136500	5.237400	1.589700	0.25235809	C	4.543800	4.469700	-2.307900	0.30696316	C	3.836100	5.705400	-0.450500	0.28576144
N	1.918000	11.031700	3.346200	-0.97452062	N	-0.423500	1.051300	-2.012900	-1.03888635	N	-0.064100	1.548200	-2.349700	-0.99098317
H	1.947990	10.736024	4.315185	0.43516180	H	-0.480031	1.052516	-3.024581	0.45695551	H	0.053671	1.792765	-3.324939	0.45424695
H	1.067542	11.574888	3.213875	0.37295569	H	-1.293777	1.444268	-1.641359	0.35803415	H	-1.021834	1.280537	-2.164670	0.39760907
H	1.682867	1.878640	1.633681	0.17090757	H	6.618712	6.886815	-3.465242	0.18697715	H	6.770314	7.384158	-0.202709	0.17615289
H	-5.831116	3.453527	2.465255	0.44503235	H	-2.736663	3.923406	2.393765	0.44837455	H	-4.175252	3.767661	2.367489	0.44793734
H	4.248564	11.877071	-6.027441	0.21201301	H	4.356258	8.649887	-4.160956	0.17865540	H	1.494395	7.969100	1.369316	0.13099042
C	1.574867	8.100704	-4.093804	0.97791716	C	2.001067	7.551065	-1.661046	0.99557902	C	-0.379392	7.217008	-2.018187	1.13379155
C	-0.082530	7.559514	-5.530680	0.96825687	C	0.067691	7.916708	-2.774692	1.00892044	C	0.191261	8.022699	-4.051494	0.93893758
C	-0.053829	6.661701	-3.456598	0.94606575	C	0.282457	6.096669	-1.452695	0.91618626	C	-0.579485	5.908792	-3.849050	1.14510691
N	1.078971	7.309309	-3.121232	-0.86528252	N	1.545889	6.409816	-1.110587	-0.90093200	N	-0.736233	6.021089	-2.518529	-1.03568486
N	1.024183	8.286208	-5.310172	-0.89583492	N	1.320401	8.339234	-2.517041	-0.89454048	N	0.095360	8.254286	-2.732623	-0.97242844
N	-0.671577	6.735736	-4.649054	-0.83393972	N	-0.512070	6.813112	-2.269289	-0.90363424	N	-0.126942	6.875119	-4.674071	-0.94025539
N	-0.675135	7.677712	-6.753744	-1.10011305	N	-0.664875	8.648588	-3.641822	-1.02071723	N	0.602933	9.064178	-4.831995	-0.90603866
N	-0.625607	5.881606	-2.512261	-1.00756926	N	-0.237609	4.973746	-0.915091	-0.90889464	N	-0.862938	4.705215	-4.396827	-1.05682508

N	2.738765	8.732969	-3.846343	-0.99361146	N	3.247514	7.936272	-1.320570	-1.01735219	N	-0.535479	7.397059	-0.688897	-1.01331082
H	-0.095457	5.657509	-1.683441	0.47454710	H	0.372608	4.335105	-0.426674	0.43908349	H	-1.350795	4.023481	-3.835206	0.47209110
H	-1.352295	5.240576	-2.793834	0.44433473	H	-1.130349	4.637443	-1.244920	0.42015425	H	-0.918442	4.627865	-5.400700	0.45940946
H	-1.279543	6.912315	-7.021220	0.47096299	H	-1.645577	8.435388	-3.752451	0.45290807	H	0.939311	8.836098	-5.757011	0.42938200
H	-0.105044	8.069259	-7.490833	0.48939855	H	-0.327847	9.552040	-3.939014	0.44491721	H	1.068807	9.827039	-4.358126	0.41206868
H	3.050498	9.453258	-4.481376	0.45560311	H	3.681513	8.726919	-1.775790	0.47749085	H	-0.181985	8.237628	-0.258454	0.44395243
H	3.117526	8.725396	-2.912345	0.45518555	H	3.834060	7.296126	-0.805253	0.44455264	H	-0.761223	6.604302	-0.107256	0.45504045

Hirshfeld surface analysis

Hirshfeld surface analysis between the two fragments, MEL template (inner fragment) and binding sites (outer fragment), is shown in Table S4.An additional C-N pair was observed due to the exposed carbon atoms of MEL interacting with the binding site through its sidewise orientation inside the cavity.

Table S3. Calculated contact area between different pairwise elements of two fragments.

**Complex**	**Binding site 1**		**Binding site 2**		**Binding site 3**	
**Element pairs (inside-outside)**	Contact area (Å^2)	Percentage (%)	Contact area (Å^2)	Percentage (%)	Contact area (Å^2)	Percentage (%)
**H-H**	87.85	**50.23**	56.93	**31.83**	79.73	**49.06**
**H-C**	18.84	10.77	44.23	24.73	15.58	9.59
**H-N**	1.51	0.87	0.25	0.14	1.30	0.80
**H-O**	6.77	3.87	7.89	4.41	7.40	4.55
**C-H**	11.49	6.57	15.49	8.66	6.07	3.73
**C-C**	2.33	1.33	1.61	0.90	3.83	2.35
**C-O**	1.43	0.82	0.44	0.25	3.95	2.43
**N-H**	**28.85**	**16.49**	**36.34**	**20.32**	**25.54**	**15.72**
**N-C**	13.30	7.60	12.02	6.72	14.82	9.12
**N-N**	0.27	0.15	0.70	0.39	0.29	0.18
**N-O**	2.27	1.30	2.95	1.65	3.77	2.32
**C-N**					0.24	0.15

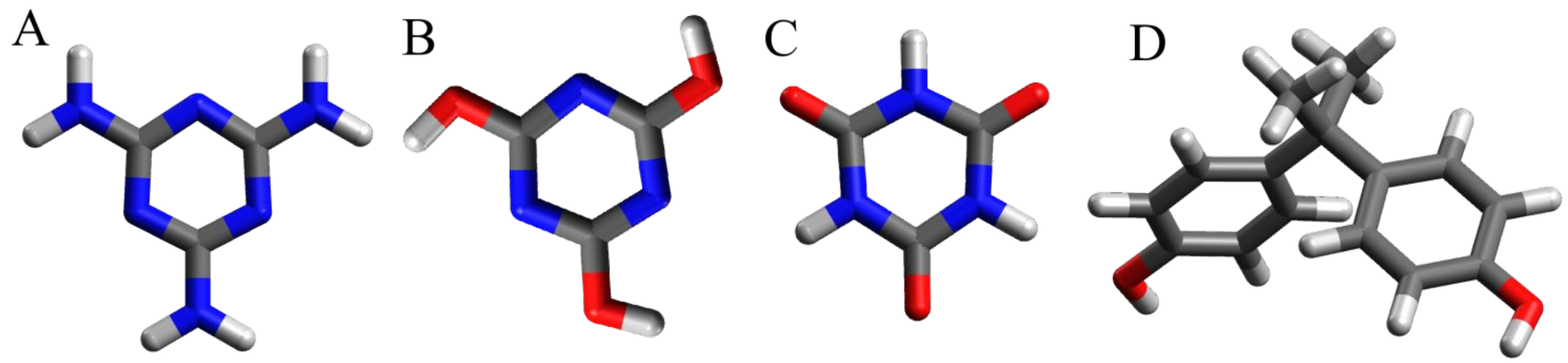

Figure S1. The optimized (r2SCAN-3c) molecules **(A)** melamine (MEL; 1,3,5-Triazine-2,4,6-triamine template, **(B)** CYA (1,3,5-triazine-2,4,6-triol tautomer), **(C)** isocyanuric acid (IsoCYA; trione tautomer) and **(D)** Bisphenol A (BPA; 4,4'-Isopropylidenediphenol in DMSO solvent made by Avogadro software.

NCI analysis

Extensive green surface area and spotted H-bonds show high affinity of PoAP-based binding site towards BPA in Figure S1 (D-F). IsoCYA bound into the binding site displays stronger H-bonds with blue isosurface but lower polar VDW interactions. Complexes B and E correspond to the bound of IsoCYA and BPA into the binding site 2 and expose the lowest interactions and partly placed molecules into the cavity.

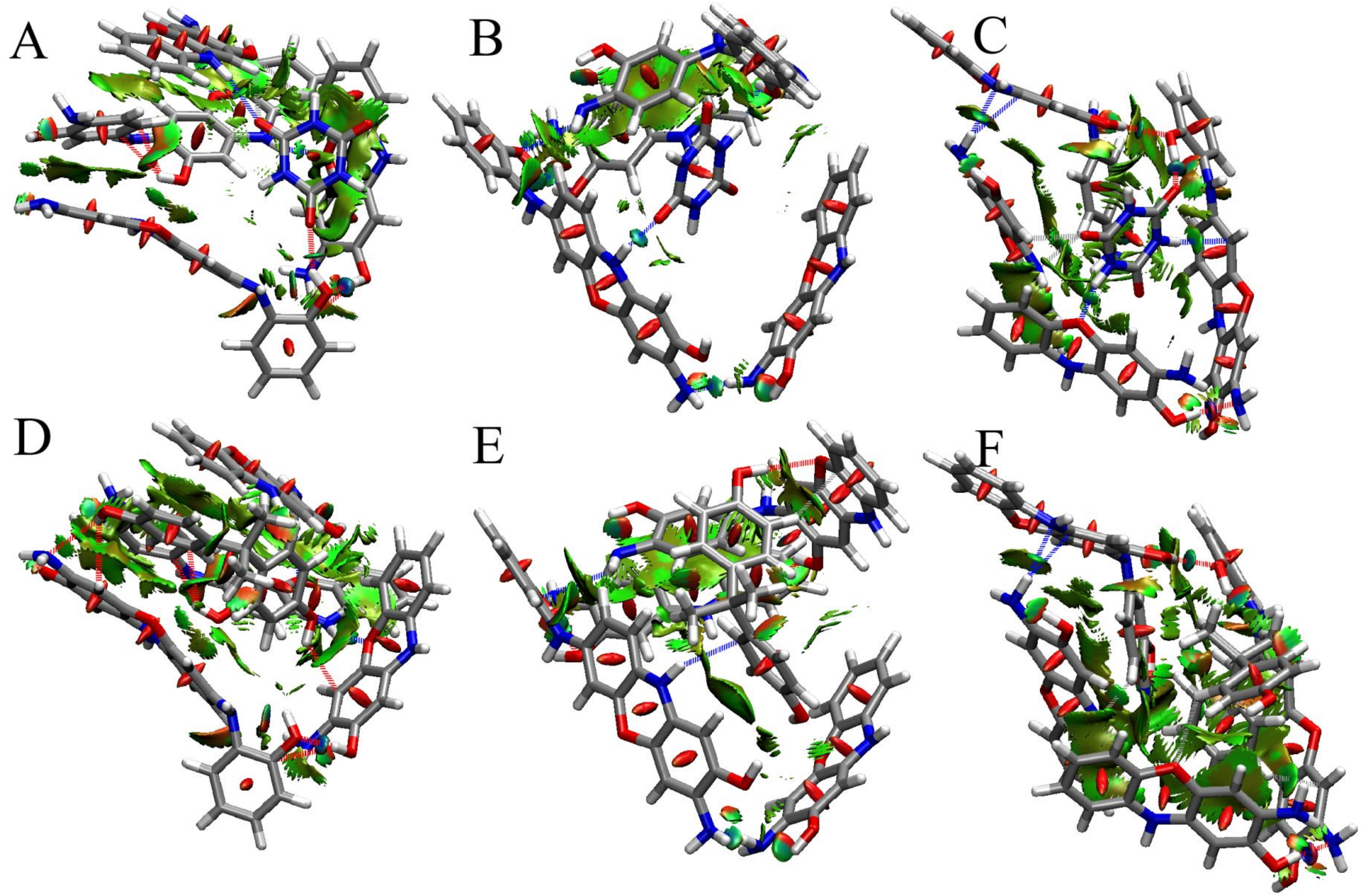

Figure S2. RDG isosurface of **(A-C)** MIP-IsoCYA in three binding sites, **(D-F)** MIP-BPA in three binding sites. The dashed lines show detected H-bonds, colored by the atom of the H-donor.

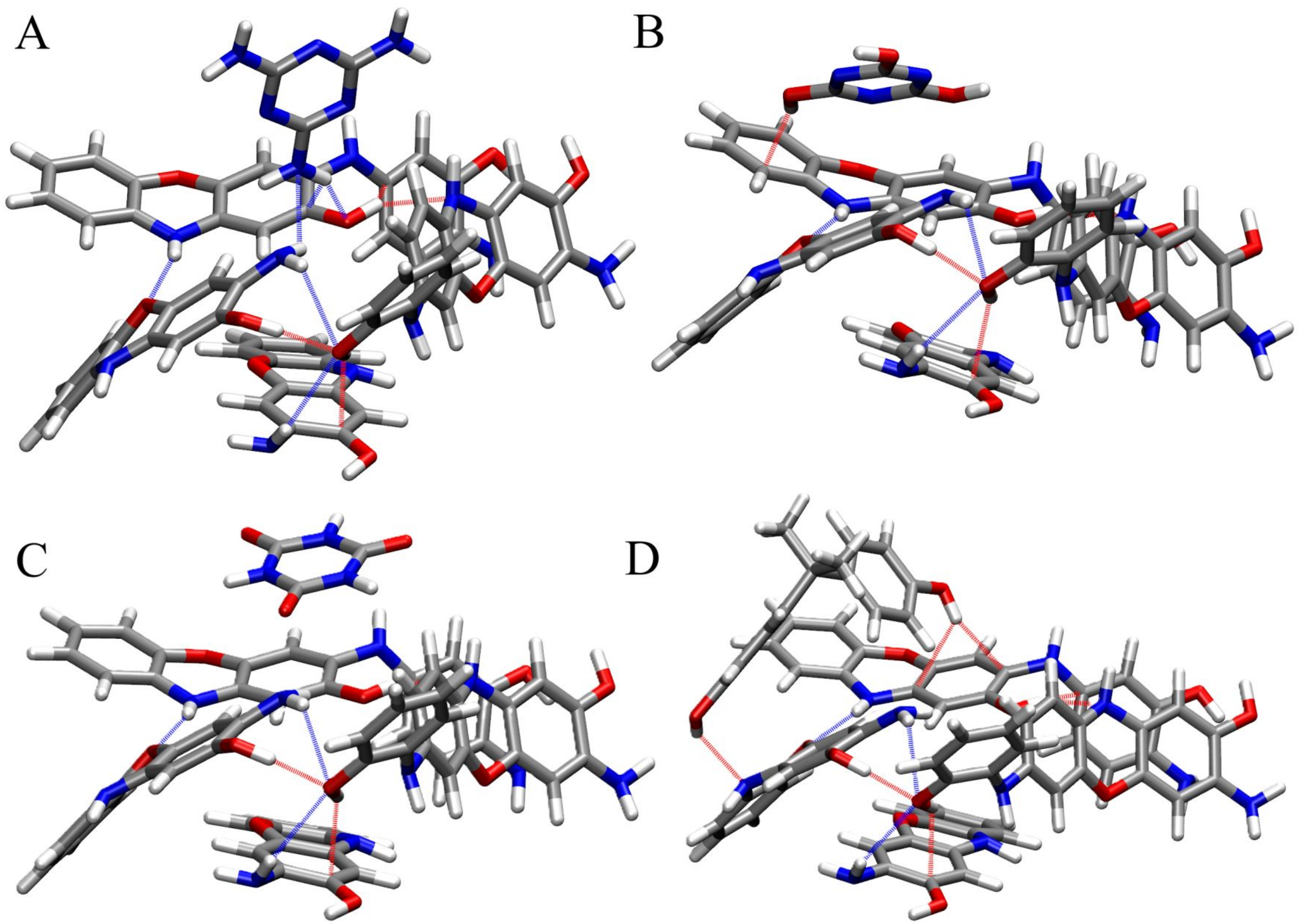

Figure S3. Non-imprinted polymer complexed with **(A)** MEL, **(B)** CYA, **(C)** IsoCYA, and **(D)** BPA, and the formed Hbonds represented in a dashed line.
